# Co–Infecting Mycoviruses VdPV1 and VdMoV1 Attenuate *Verticillium dahliae* and Are Transmitted Vertically and Horizontally

**DOI:** 10.3390/v18070795

**Published:** 2026-07-19

**Authors:** Yifan Wang, Guolong Gao, Jiafeng Huang, Shicheng Wang, Hongyu Ji, Yukun Liu, Yejuan Du

**Affiliations:** 1 Key Laboratory of Oasis Agricultural Pest Management and Plant Protection Resources Utilization, College of Agriculture, Shihezi University, Shihezi 832003, China; 2 College of Agriculture, Shihezi University, Shihezi 832003, China

**Keywords:** mycoviruses, *Verticillium dahliae*, hypovirulence, co–infection

## Abstract

Cotton verticillium wilt caused by *Verticillium dahliae* severely restricts global cotton production. Mycovirus–induced hypovirulence provides a promising strategy for sustainable disease management. In this study, *V. dahliae* isolates from Xinjiang were screened by virome sequencing and RT–PCR, and strain 121–11C–1 co–infected with Verticillium dahliae partitivirus 1 (VdPV1) and Verticillium dahliae magoulivirus 1 (VdMoV1) was identified. Virus–free isogenic strains were obtained via single–conidium purification, verifying that both viruses can be vertically transmitted through fungal conidia. Dual–culture assays uncovered a novel, previously unreported horizontal transmission dependency between the two viruses: VdPV1 achieves horizontal transmission only with the assistance of VdMoV1, whereas VdMoV1 can transmit independently or co–transmit with VdPV1. Biological and pathogenicity assays demonstrated that both viruses significantly inhibit mycelial growth, reduce conidial production and attenuate fungal virulence. Notably, dual infection induced a significantly stronger hypovirulent phenotype than single infection. These findings elucidate the transmission dynamics of VdPV1 and VdMoV1, expand the repertoire of hypovirulent mycoviruses in *V. dahliae*, and provide evidence that viral co–infection enhances fungal attenuation, offering potential applications for biological control of cotton Verticillium wilt.

## 1. Introduction

As one of the most important cash crops worldwide, cotton serves as a key raw material for the textile industry due to its excellent air permeability and moisture absorption properties. Its production plays a critical role in agricultural economies, rural employment, and international trade [1,2,3]. However, cotton production is severely constrained by Verticillium wilt, a destructive soil–borne disease caused by the phytopathogenic fungus *Verticillium dahliae*, which leads to substantial yield losses and remains difficult to control because of the long–term survival of microsclerotia in soil and the broad host range of the pathogen [4,5].

Mycoviruses are a diverse group of viruses that infect fungi and replicate within fungal cells. Since their first discovery in cultivated mushrooms by Hollings in 1960, mycoviruses have attracted increasing attention due to their widespread distribution and diverse biological effects on fungal hosts [6,7]. Depending on virus–host interactions, mycoviral infections can be broadly categorized into hypovirulent, neutral, or mutualistic infections [8]. Mycovirus–fungus mutualism entails no negative impact on the host but instead confers clear benefits, including improved stress resistance and hypervirulence [9]. For example, infection of Trichoderma harzianum with Trichoderma harzianum mycovirus 1 (ThMV1) enhances the host’s biocontrol capacity and salt stress tolerance, directly demonstrating the positive regulation of host beneficial traits by the virus and achieving virus–host mutualism [10]. Neutral viruses are characterized by asymptomatic host phenotypes [11]. In most cases, mycovirus infection causes little or no symptoms in the host. For example, Fusarium graminearum gemytripvirus 1 (FgGMTV1) does not significantly affect the host fungal phenotype [12]. Such interactions reflect a balanced strategy resulting from long–term co–evolution between viruses and their fungal hosts, and they represent an important basis for the widespread distribution of mycoviruses in nature [13,14].

The hypovirulent trait remains a major research focus and the central rationale for using mycoviruses in biological control. The successful management of chestnut blight in Europe serves as a classic example: by artificially releasing CHV1–carrying hypovirulent strains into cankers on infected trees, the fungus exhibits reduced pigmentation, decreased oxalic acid accumulation, altered extracellular enzyme activities, and significantly attenuated virulence [15,16,17,18].

Based on genome type and replication strategy, mycoviruses are classified into several major groups, including double–stranded RNA (dsRNA) viruses, positive–sense single–stranded RNA (+ssRNA) viruses, negative–sense single–stranded RNA (–ssRNA) viruses, reverse–transcribing RNA viruses, and single–stranded DNA (ssDNA) viruses. According to the latest taxonomy released by the International Committee on Taxonomy of Viruses (ICTV, 2025) (https://ictv.global/), a total of 41 viral families have been formally established, highlighting their remarkable diversity and evolutionary complexity [19,20,21]. Among them, the families *Partitiviridae* and *Botourmiaviridae* are particularly relevant in this study.

The family *Partitiviridae* comprises double–stranded RNA (dsRNA) genomes. Members of this family are widely distributed among fungi, plants, and protists and represent one of the most diverse groups of mycoviruses, which can be classified into five genera: *Alphapartitivirus*, *Betapartitivirus*, *Gammapartitivirus*, *Deltapartitivirus*, and *Cryspovirus* [22,23]. Several genera, including *Alphapartitivirus*, *Betapartitivirus*, and *Gammapartitivirus*, have been reported to contain hypovirulent strains that reduce host growth rate, sporulation, and pathogenicity. These characteristics make partitiviruses important candidates for the biological control of plant pathogenic fungi such as *Sclerotinia sclerotiorum*, *Rhizoctonia solani*, and *Botryosphaeria dothidea* [24,25]. 

In contrast, the family *Botourmiaviridae* comprises positive–sense single–stranded RNA viruses that infect both plants and fungi. This family is taxonomically divided into multiple genera, among which only *Ourmiavirus* infects plants, while the remaining genera predominantly infect fungi [26,27]. With the rapid development of high–throughput sequencing technologies, increasing numbers of botourmiaviruses have been identified in diverse fungal hosts, including *Magnaporthe oryzae*, *Colletotrichum camelliae*, and *Botryosphaeria dothidea*, further expanding the known diversity of fungal RNA viruses [28,29,30,31].

Unlike plant and animal viruses, most mycoviruses lack movement proteins for intercellular transport. Therefore, they persist in fungal populations primarily through vertical transmission and horizontal transmission [9,19,32]. Vertical transmission occurs when viral particles are incorporated into fungal conidia or other asexual spores and subsequently inherited by progeny during germination [33,34,35]. Horizontal transmission requires hyphal contact between compatible strains, allowing viral movement through cytoplasmic exchange [36]. This process is often restricted by vegetative incompatibility systems, which trigger programmed cell death upon fusion between genetically distinct strains, thereby limiting viral spread in fungal populations [37,38,39]. 

To date, several mycoviruses have been identified in *V. dahliae*, including Verticillium dahliae partitivirus 1 (VdPV1), Verticillium dahliae chrysovirus 1 (VdCV1), and Verticillium dahliae magoulivirus 1 (VdMoV1). Among these, VdPV1 is a dsRNA virus with a typical two–segment genome encoding RNA–dependent RNA polymerase (RdRp) and coat protein (CP), whereas VdMoV1 is a +ssRNA virus belonging to *Botourmiaviridae*. Although the genomes of these viruses have been characterized, their biological roles in regulating fungal growth, development, and pathogenicity remain insufficiently understood. In particular, the effects of viral co–infection and their transmission characteristics in *V. dahliae* have not been systematically investigated. Therefore, in this study, we identified a *V. dahliae* strain co–infected with VdPV1 and VdMoV1 through virome sequencing and RT–PCR validation. We further constructed virus–cured and virus–transmitted derivative strains to investigate (i) the transmission modes of the two mycoviruses, (ii) their effects on fungal biological traits and pathogenicity, and (iii) the potential interactions between co–infecting viruses. The results provide new insights into mycovirus–host interactions and offer potential viral resources for the biological control of cotton Verticillium wilt.

## 2. Materials and Methods

### 2.1. Verticillium dahliae Strains and Cultural Conditions

The *Verticillium dahliae* strains used in this experiment were previously isolated, identified and preserved by our laboratory. Strain 01611 is a *V. dahliae* strain with G418 resistance constructed in the laboratory, which has been identified as a highly virulent strain [40] and served as the control in this study. All strains were cultured on PDA medium at 26 °C in darkness for 15 days. Subsequently, colony morphology was observed, and the cultural characteristics of *V. dahliae* were classified. According to the quantity of microsclerotia of the colonial morphology on PDA, the strains were divided into three cultural types: the hyphal type, the intermediate type and the sclerotium type [41].

### 2.2. Metavirome Sequencing

Focus on strains with abnormal phenotypes and slow growth rates from the three morphological types (mycelial, intermediate, and sclerotial) respectively for mixed–sample sequencing. In total, 13 strains belonged to the mycelial type, 6 to the intermediate type, and the sclerotial type was further divided into sclerotial type I (6 strains) and sclerotial type II (8 strains). For each morphological type, place mycelial plugs of the selected strains into Czapek–Dox medium under constant agitation (200 rpm) at 26 °C in complete darkness. The mycelial pellets were collected, rapidly frozen in liquid nitrogen, and stored on dry ice before being sent to Qingdao Oruijin Biotech Co., Ltd (Qingdao, China) for sequencing. The obtained sequences were aligned and assembled using DNAMAN 9 software and the NCBI database, followed by screening to identify fungal mycovirus sequences.

### 2.3. RNA Extraction and RT–PCR Detection

The mycelial pellet collected in Section 2.2. was frozen in liquid nitrogen, transferred to a mortar, and thoroughly ground. The ground powder was then placed into a 1.5 mL nuclease–free centrifuge tube, and total RNA was extracted using Vazyme FreeZol Reagent (Vazyme, Nanjing, China). Specific primers were designed using Primer Premier 6 software based on the obtained mycovirus contigs (Supplementary Table S1). Subsequently cDNA was synthesized using specific primers with Vazyme HiScript IV RT SuperMix for qPCR (+gDNA wiper). RT–PCR was performed with specific primers to detect viruses in *V. dahliae* strain. The RT–PCR reaction mixture consisted of 1 µL cDNA, 0.4 µL forward primer, 0.4 µL reverse primer, 5 µL Dongsheng 2× PCR Mix, and 3.2 µL ddH_2_O. The reaction procedure was as follows: pre–denaturation at 94 °C for 3 min; denaturation at 94 °C for 30 s; annealing at 55 °C for 30 s; extension at 72 °C for 16 s. A total of 30 cycles were performed starting from the denaturation step, followed by a final extension at 72 °C for 10 min. The amplified products were cloned into the pMD™19–T vector using a cloning kit (Takara, Dalian, China) and subsequently sequenced. Subsequently, BLASTx searches were performed in NCBI to analyze sequence homology.

### 2.4. Virus Curing

Virus elimination was performed via single–spore isolation combined with ribavirin treatment at a concentration of 100 µg/mL and hyphal tip culture. The hyphal tips of virus–infected strains were collected and transferred into centrifuge tubes containing 1 mL of sterile water. After thorough mixing, 100 µL of the suspension was transferred into a new sterile centrifuge tube supplemented with 900 µL of sterile water for gradient dilution, and this dilution step was repeated three times. Subsequently, 100 µL of the final dilution (1000–fold dilution) was spread onto PDA medium supplemented with ribavirin for incubation to obtain conidial progenies. After 10 generations of serial subculture of hyphal tips harvested from single conidial progenies, RT–PCR was carried out for viral screening.

### 2.5. Viral Horizontal Transmission Assay

A dual culture method was adopted: the virus-infected strain 121–11C–1 was inoculated on one side of a PDA plate. After 4 days of incubation, the virus–free *Verticillium dahliae* strain 01611^G418^ was inoculated on the opposite side of the PDA plate, approximately 1 cm away from the virus–infected strain. Five replicates were set for each strain. Following incubation at 26 °C for 20 days or longer, mycelial plugs were taken from the hyphal contact zone and transferred onto PDA medium supplemented with G418 for further culture. After the colonies emerged and were subcultured for three generations, the virus presence in the recipient strains was detected.

### 2.6. Characterization of Strain Biological Traits

Mycelial plugs of each strain were inoculated in the center of PDA medium to determine the mycelial growth rate and observe colony morphology. The colony diameter was measured and recorded for each isolate at intervals of 5–9dpi. Colony morphology was observed on the 15th day. Each strain was set up with five replicates. The growth rate (mm/d) was calculated as follows: Growth rate = (Diameter on day 9 – Diameter on day 5)/4 [42].

Mycelia of each strain were streaked and cultured on PDA medium using sterile toothpicks. Meanwhile, sterilized coverslips were obliquely inserted into the streaked areas of the PDA medium. After 3 days of incubation, the mycelial morphology and conidiophores stalks were observed under an OLYMPUS microscope, with three replicates set for each strain [43].

Pipette 200 µL of spore suspension adjusted to a concentration of 10^6^ conidia/mL, and inoculate it into 100 mL of Czapek’s medium for shaking culture at 26 °C and 200 rpm/min. The conidial production was observed and recorded from day 3 to day 7 of incubation. Each treatment was performed in three biological replicates per strain [43].

Pipette 200 μL of conidial suspension with a concentration of 10^6^ conidia/mL and spread it evenly on BMM medium covered with cellophane. After dark incubation at 26 °C for 15 days, the microsclerotia morphology was observed under a microscope, and the fresh and dry weights of microsclerotia were measured [43].

In order to evaluate the penetration ability of the tested strains, mycelial of each strain were inoculated onto MM medium covered with cellophane using sterile toothpicks. After 3 days of incubation, the cellophane layers were removed, and the cultures were continuously incubated in darkness at 26 °C until the 7th day. The mycelial growth on the underlying medium was observed to determine whether the hyphae could penetrate the cellophane [43].

### 2.7. Pathogenicity Assay

Cotton cultivar 108 fu was selected as the test plant for pathogenicity assays. When cotton seedlings grew to the 3–4 true–leaf stage, pathogenicity identification was performed via the root–dip inoculation method. 200 mL of conidial suspension at a concentration of 10^6^ conidia/mL was added to each hydroponic box. After 30 min of immersion inoculation, the suspension was discarded, and the cotton roots as well as hydroponic boxes were thoroughly rinsed with clean water. The disease index was observed and recorded starting one week after inoculation, with data collected every two days. Disease severity was evaluated according to the standardized grading criteria for cotton Verticillium wilt, and the disease index was calculated by Shao, et al. [44]. At 30 days post inoculation (dpi), cotton stems were longitudinally dissected to observe vascular browning. For each strain, five hydroponic boxes of cotton seedlings were inoculated, with 12 seedlings per box, resulting in a total of 60 seedlings for each treatment.

### 2.8. Statistical Analysis

All data are presented as mean ± standard deviation. Statistical analyses were performed using GraphPad Prism (10.1.2). Two–way analysis of variance (two–way ANOVA) was applied for colony diameter, conidial production, fresh weight and dry weight of microsclerotia, while one–way analysis of variance (one–way ANOVA) was used for the average growth rate.

## 3. Results

### 3.1. Co–Detection of VdPV1 and VdMoV1 in Strain 121–11C–1

**Figure 1:**
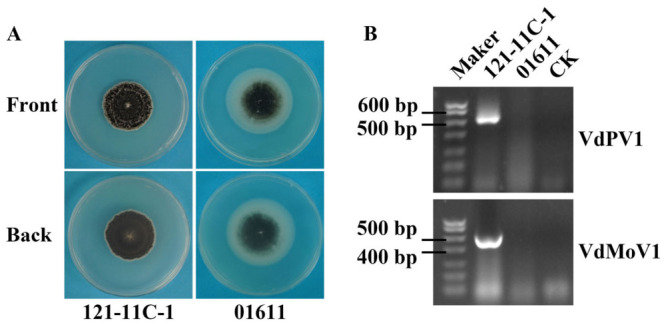
Identification of virus–infected strain 121–11C–1. (**A**) Colony morphology of strains 121–11C–1 and 01611. (**B**) RT–PCR detection of VdPV1 and VdMoV1.

Based on colony phenotypic characteristics, the tested strains can be divided into three major types: mycelial type, intermediate type, and sclerotial type. The mycelial type (VS) is characterized by abundant mycelia, no production of black microsclerotia, and white colonies; the intermediate type (VM) produces a small number of microsclerotia in the center of the colony; the sclerotial type (VH) produces a large number of black microsclerotia, with inconspicuous mycelia and black colonies. To explore potential mycoviral diversity associated with abnormal fungal phenotypes, 33 *V. dahliae* strains representing different morphological types (mycelial, intermediate, and microsclerotial) were subjected to metavirome sequencing. A total of 21 mycoviruses–related contigs were identified through BLAST searches against the NCBI database (Appendix A). Among these, five contigs (contigs 4838, 4530, 5990, 5676, and 4525) belonging to the family *Partitiviridae* exhibited less than 90% sequence identity at the nucleotide level to any previously characterized partitiviruses, indicating that they represent novel viral species. Specifically, contigs 4838, 4530, and 5990 share overlapping regions and were collectively designated as Verticillium dahliae partitivirus 2 (VdPV2) (Supplementary Figure S1, Supplementary Table S2), whereas contigs 5676 and 4525, which also contain overlapping segments, were tentatively named Verticillium dahliae partitivirus 3 (VdPV3) (Supplementary Figure S2, Supplementary Table S2). Additionally, four contigs (19074, 19083, 5397, and 7674) showed high similarity to Verticillium dahliae partitivirus 1 (VdPV1), also a member of the family *Partitiviridae*. Seven other contigs (15097, 15987, 17699, 19940, 20798, 20854, and 9403) were assigned to Verticillium dahliae magoulivirus 1 (VdMoV1) within the family *Botourmiaviridae*, while the remaining five contigs (11639, 9446, 10957, 6649, and 6965) displayed homology to Lentinula edodes deltaflexivirus 2 (LeDF2) in the family *Deltaflexiviridae* (Supplementary Table S2). To validate sequencing results, RT–PCR assays were performed on candidate strains. The microsclerotial strain 121–11C–1 was confirmed to be co–infected with both VdPV1 and VdMoV1, whereas other viruses were not stably detected by RT–PCR due to their low abundance (Figure 1 and Figure S1).

### 3.2. Establishment of Virus–Cured Derivatives from Co–Infected Strain 121–11C–1

**Figure 2:**
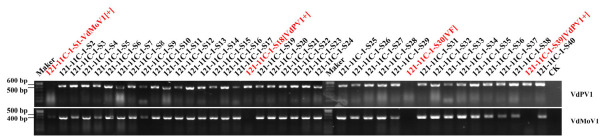
RT–PCR detection of VdPV1 and VdMoV1 in monoconidial progenies of strain 121–11C–1. Water was used as a negative control (CK), and a 700 bp DNA ladder was used as the size marker. The expected amplicon sizes were 519 bp for VdPV1 and 439 bp for VdMoV1.

Virus elimination was performed using a combination of single–conidium isolation, ribavirin treatment, and hyphal tip culture. A total of 40 monoconidial derivatives were obtained and screened for viral presence. RT–PCR analysis revealed heterogeneous virus elimination patterns among progeny strains. Specifically, strain S1 was cured of VdPV1, whereas strains S18 and S39 were cured of VdMoV1. Notably, strain S30 was completely virus–free, lacking both VdPV1 and VdMoV1 (Figure 2). Overall, the elimination rates were low (2.5–5% for individual viruses), and only 2.5% of progeny strains were fully virus–free, with the total virus elimination rate reaching merely 10%. These results indicate that both VdPV1 and VdMoV1 are efficiently transmitted through conidia and are stably maintained in the fungal host.

### 3.3. Effects of Co–Infection with VdPV1 and VdMoV1 on Biological Characteristics

**Figure 3:**
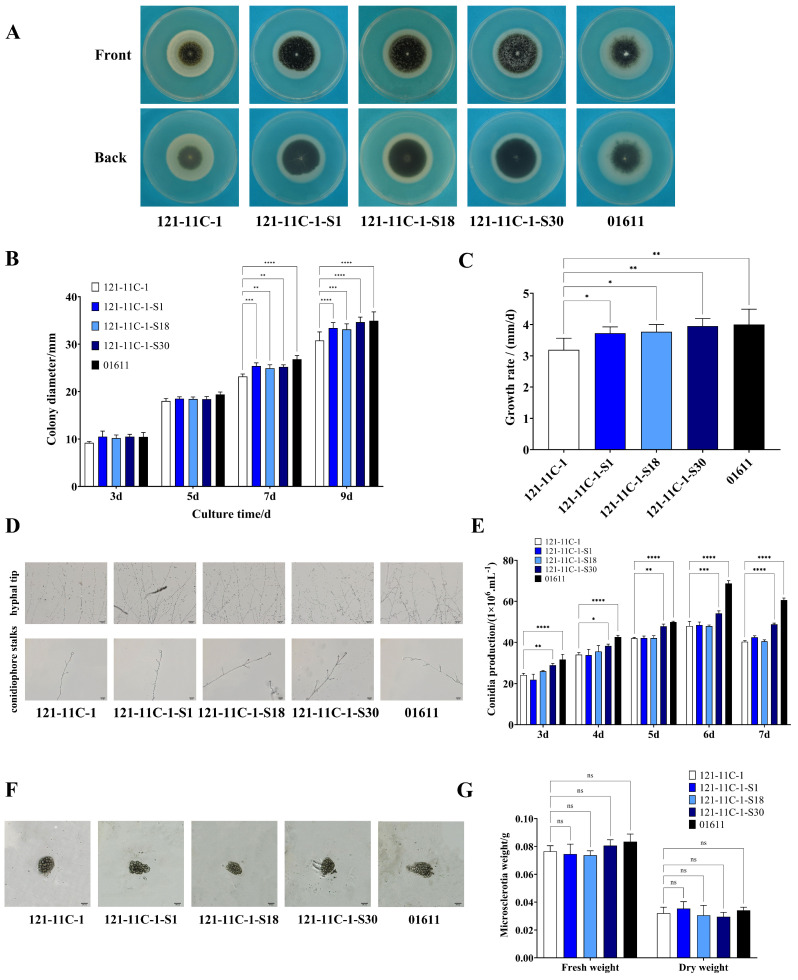
Biological traits of co–infected strain 121–11C–1 and its virus–cured strains. (**A**) Colony morphology. Colonies of strain 121–11C–1 and its virus–cured derivatives (121–11C–1–S1, 121–11C–1–S18, 121–11C–1–S30) were cultured on PDA medium at 26 °C for 15 days. (**B**) Colony diameter measured on days 3, 5, 7 and 9. Data are presented as mean ± standard deviation (*n* = 10). Two–way ANOVA was used for statistical analysis. (**C**) Average growth rate. Data are presented as mean ± standard deviation (*n* = 10). One–way ANOVA was used for statistical analysis. (**D**) Microscopic observation of hyphal tips and conidiophores. Scale bar for hyphal tips = 100 µm; scale bar for conidiophores = 20 µm. (**E**) Conidial production determined during days 3–7 of cultivation. Data are presented as mean ± standard deviation (*n* = 3). Two–way ANOVA was used for statistical analysis. (**F**) Microscopic observation of microsclerotia morphology. Scale bar = 20 µm. (**G**) Fresh and dry weights of microsclerotia. Data are presented as mean ± standard deviation (*n* = 5). Two–way ANOVA was performed. ns: not significant, * *p* < 0.05, ** *p* < 0.01, *** *p* < 0.001, **** *p* < 0.0001.

To clarify the effects of VdPV1 and VdMoV1 on the biological characteristics of *V. dahliae*, we determined the biological traits of three derivatives: strain S1 (cured of VdPV1), strain S18 (cured of VdMoV1) and fully virus–free strain S30. The measurement of average growth rate revealed that all virus–cured strains and the control strain exhibited significantly higher growth rates than the co–infected strain 121–11C–1 (Figure 3A–C). Observations on mycelial morphology and conidiophore stalks revealed that the co–infected strain 121–11C–1 and partially cured strains S1 and S18 presented sparse mycelial growth, low mycelial density, and reduced branching of conidiophores. In contrast, the fully virus–cured strain S30 and the virus–free control strain 01611 showed dense and compact mycelia, as well as highly developed conidiophores with abundant branches (Figure 3D). Conidial production further indicated that there was no significant difference between the co–infected strain 121–11C–1 and partially cured strains S1 and S18, while their conidial production was significantly lower than those of the fully cured strain S30 and the control strain 01611 (Figure 3E). In addition, no significant differences in microsclerotium fresh weight and dry weight were detected among all strains, suggesting that the two mycoviruses had no obvious influence on microsclerotium formation (Figure 3F,G).

In summary, co–infection with VdPV1 and VdMoV1 markedly inhibits the colony growth rate, mycelial morphology, conidiophore development, and conidial production of *V. dahliae*. Although partially virus–cured strains exhibited a certain recovery in mycelial growth rate compared with the co–infected strain, no significant differences in other biological traits were observed, indicating that partially cured strains still retained hypovirulent traits. The biological traits of fully virus–cured strains were highly similar to those of the virus–free control strain.

### 3.4. Hypovirulence Conferred by Co–Infection of VdPV1 and VdMoV1

**Figure 4:**
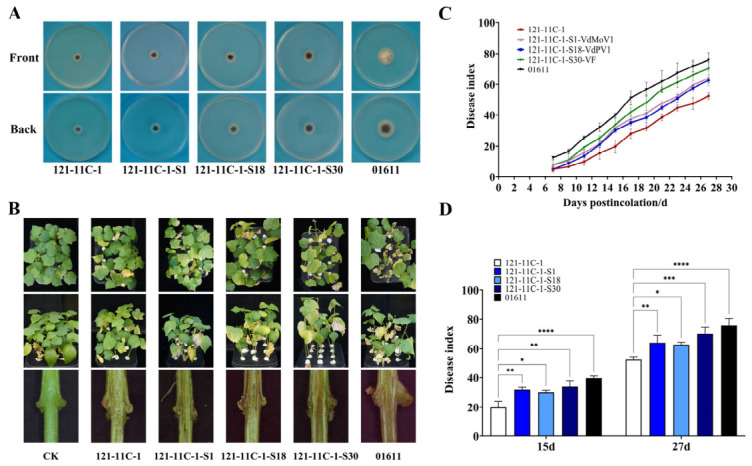
Pathogenicity assay of the co–infected strain 121–11C–1 and its virus–cured strains. (**A**) Penetration ability of tested strains on MM medium. (**B**) Disease symptoms and vascular browning of cotton plants at 30 days post inoculation. (**C**) Dynamic changes in disease index of cotton plants from 0 to 27 days post inoculation. (**D**) Significance analysis of disease index at 15 and 27 days post inoculation. All data are presented as mean ± standard deviation. Statistical significance was evaluated using two–way ANOVA. Three biological replicates were performed (*n* = 3), and 60 cotton plants were inoculated for each strain (N = 60). ns: not significant, * *p* < 0.05, ** *p* < 0.01, *** *p* < 0.001, **** *p* < 0.0001.

To further clarify the effects of VdPV1 and VdMoV1 on the pathogenicity of the host fungus, the penetration capacity of each strain was determined first. The results showed that the hyphae of all strains could penetrate cellophane and grow on the underlying medium, indicating that the presence or absence of mycoviruses did not impair the basic penetration ability of *V. dahliae* strains (Figure 4A). Subsequently, cotton seedlings were inoculated with conidial suspension to explore the influence of mycoviruses on fungal pathogenicity. Pathogenicity assays revealed that the co–infected strain 121–11C–1 exhibited the weakest virulence, with fewer yellowed and wilted leaves and the lowest disease index. The partially virus–cured strains S1 and S18 presented a significant recovery in pathogenicity, accompanied by aggravated leaf yellowing and wilting, and their disease indices were markedly higher than those of strain 121–11C–1. The fully virus–cured strain S30 showed a further recovery in virulence, which was close to but slightly lower than that of the virus–free control strain 01611. The virus–free control had the highest disease index and the strongest pathogenicity (Figure 4B–D). Longitudinal section observation of cotton stems showed that cotton plants inoculated with strain 121–11C–1 displayed the mildest vascular browning. Vascular discoloration was aggravated in cotton plants infected with partially cured strains S1 and S18, while the most severe vascular browning was observed in plants inoculated with the fully cured strain S30 and the control strain (Figure 4B).

### 3.5. Horizontal Transmission of VdPV1 and VdMoV1 Under Co–Infection

**Figure 5:**
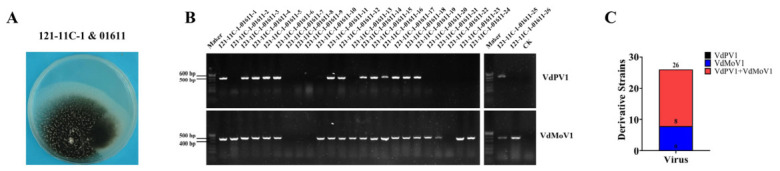
Horizontal transmission determination of VdPV1 and VdMoV1. (**A**) Dual culture of strain 121–11C–1 and virus–free strain 01611. (**B**) RT–PCR detection of VdPV1 and VdMoV1 in derivatives of strain 01611. (**C**) Statistics of viral carriage in derivatives of strain 01611.

To examine the horizontal transmission capacity of the two viruses under the coinfection condition of the donor strain, dual culture assays were performed between donor strain 121–11C–1 and recipient strain 01611. Among 26 recipient derivatives, viral transmission was successfully detected in all recipient derivatives (Figure 5). Specifically, 8 derivatives carried only VdMoV1 (30.8%), while 18 derivatives were co–infected with both VdPV1 and VdMoV1 (69.2%). Notably, no strain carrying VdPV1 alone was detected. These results indicate that VdMoV1 can be transmitted independently, whereas VdPV1 transmission is strictly dependent on the presence of VdMoV1 under co–infection conditions.

### 3.6. Mycovirus Acquisition Induces Hypovirulence in Recipient Strains

**Figure 6:**
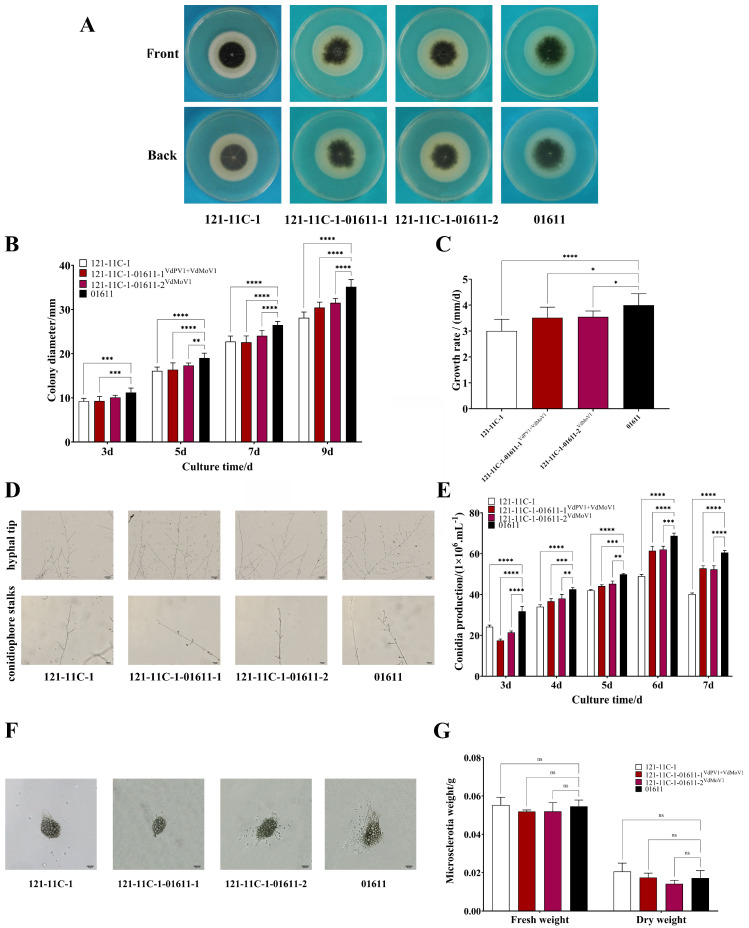
Determination of the horizontal transmission ability of VdPV1 and VdMoV1 and biological traits of virus–transmitted derivative strains. (**A**) Colony morphology of derivatives derived from strain 01611. (**B**) Colony diameter measured on days 3, 5, 7 and 9. (**C**) Average growth rate. (**D**) Microscopic observation of hyphal morphology and conidiophores. (**E**) Determination of conidial production. (**F**) Microscopic observation of microsclerotia morphology. (**G**) Measurement of fresh and dry weights of microsclerotia. ns: not significant, * *p* < 0.05, ** *p* < 0.01, *** *p* < 0.001, **** *p* < 0.0001.

To evaluate the phenotypic effects of horizontal virus transmission, two representative recipient strains were analyzed: 01611–1 (co–infected with VdPV1 and VdMoV1) and 01611–2 (infected with VdMoV1 only). Both virus–transmitted strains exhibited significantly reduced colony growth rates compared with the virus–free strain 01611 (Figure 6A–C). Microscopic examination revealed sparse hyphal structures and reduced conidiophore development in infected derivatives (Figure 6D). Conidiation assays showed a marked reduction in spore production in both infected strains throughout the observation period (Figure 6E). Although a partial recovery in sporulation was observed at later stages, values remained significantly lower than those of the virus–free control. Microsclerotia production remained largely unaffected across all strains, with no significant differences in fresh or dry weight (Figure 6F–G).

Collectively, these results indicate that horizontally acquired VdPV1 and VdMoV1 infections consistently suppress vegetative growth and sporulation, while exerting limited effects on microsclerotial development.

### 3.7. Pathogenicity of Virus–Transmitted Strains

**Figure 7:**
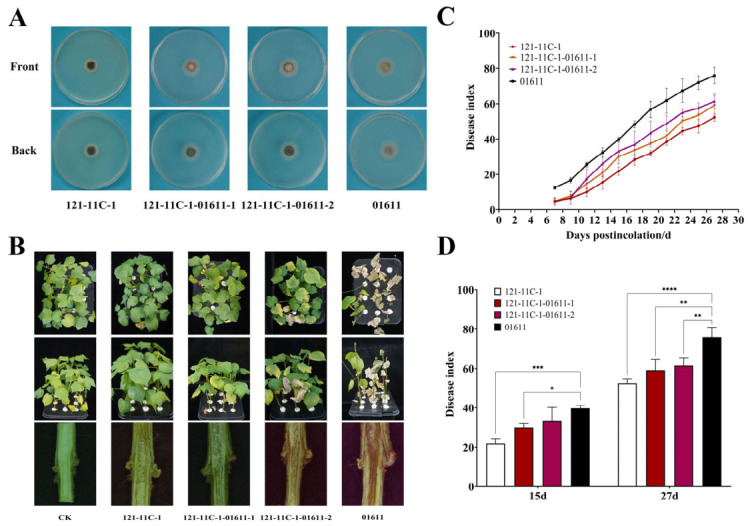
Pathogenicity assay of virus–transmitted derivative strains. (**A**) Penetration ability of strain 121–11C–1, derivative strains 121–11C–1–01611–1, 121–11C–1–01611–2 and control strain 01611 on MM medium; (**B**) Disease symptoms and vascular browning of cotton plants at 30 days post inoculation; (**C**) Dynamic changes in disease index of cotton plants from 0 to 27 days post inoculation; (**D**) Significance analysis of disease index at 15 and 27 days post inoculation. ns: not significant, * *p* < 0.05, ** *p* < 0.01, *** *p* < 0.001, **** *p* < 0.0001.

Pathogenicity assays on cotton seedlings demonstrated that virus–infected derivatives exhibited significantly reduced virulence compared with virus–free strain 01611 (Figure 7). Both 01611–1 (dual infection) and 01611–2 (single infection) induced milder disease symptoms and lower disease indices throughout the infection period. Among them, the dual–infected strain showed the weakest pathogenicity, whereas the single–infected strain displayed an intermediate phenotype (Figure 7B–D). Vascular discoloration analysis further confirmed these trends, with the most severe browning observed in virus–free infections and progressively milder symptoms in virus–infected treatments. This gradient pattern is consistent with the pathogenicity trend observed in the virus–cured strain series, establishing a bidirectional validation of “virus acquisition → reduced pathogenicity” and “virus curing → restored pathogenicity”, which strongly supports the conclusion that the viruses suppress host pathogenicity.

## 4. Discussion

Metavirome sequencing has become an indispensable approach for uncovering hidden viral diversity in fungi and has significantly advanced our understanding of mycovirus–host interactions. Compared with conventional virus detection methods, high–throughput sequencing enables the simultaneous identification of both dominant and low–abundance viral components, thereby providing a more comprehensive view of fungal viromes [45]. In this study, metavirome sequencing of *Verticillium dahliae* strains with abnormal phenotypes led to the identification of multiple viral contigs, among which VdPV1 and VdMoV1 were the most abundant. These findings highlight the utility of virome–based screening in prioritizing biologically relevant viruses potentially associated with altered fungal phenotype [46].

The establishment of a virus–cured strain system further demonstrated that both VdPV1 and VdMoV1 can be vertically transmitted through conidia, albeit with low elimination efficiency. This observation indicates that both viruses are stably maintained within fungal populations, a characteristic that is critical for their potential application as biological control agents. Stable vertical transmission ensures long–term persistence of hypovirulence traits in fungal lineages, which is a prerequisite for field–level disease suppression [36,47].

Horizontal transmission refers to the spread of mycoviruses via hyphal anastomosis. However, this process is restricted by the non self–recognition response among fungi; most mycoviruses can only transmit between vegetatively compatible strains [16]. In this study, horizontal dual–culture assays demonstrated that the virus–coinfected strain 121–11C–1 could transmit both viruses to the recipient strain 01611, confirming the horizontal transmissibility of this virus combination between the tested strains. In filamentous fungi, horizontal virus transmission generally depends on hyphal anastomosis, which is strictly governed by vegetative compatibility groups (VCGs) [48]. Therefore, to further elucidate the underlying mechanisms of virus transmission, future investigations could be extended in the following directions: (1) systematically determine the VCG types of both donor and recipient strains and conduct dual–culture experiments using strains from different, known–incompatible VCGs, so as to examine whether the viruses can indeed overcome compatibility barriers; (2) co–culture the donor strain with fungal species other than *Verticillium dahliae* (e.g., non–*V. dahliae* fungi) to evaluate the host–range breadth of this virus complex and its potential ecological risks to non–target fungi; and (3) combine fluorescent labeling with microscopic observation to track the transfer pathways of viral particles at hyphal contact interfaces or in the culture medium, thereby achieving a more precise understanding of the transmission process [49].

A key finding of this study is the asymmetric horizontal transmission behavior of the two viruses under co–infection conditions. VdMoV1 was capable of independent transmission, whereas VdPV1 required the presence of VdMoV1 for successful horizontal spread. This phenomenon may suggest a potential helper–like relationship between the two viruses, where VdMoV1 may facilitate the cellular or molecular conditions required for VdPV1 transmission. One possible explanation is that VdMoV1 may modulate host vegetative incompatibility responses or suppress antiviral defense mechanisms, thereby promoting the entry or stability of VdPV1 in recipient strains. Similar helper–dependent viral transmission strategies have been reported in other fungal virus systems, where co–infecting viruses modulate host compatibility or intracellular trafficking to enhance transmission efficiency [50,51]. This property is particularly important for biological control applications, as efficient horizontal transmission is essential for the dissemination of hypovirulence traits within pathogen populations [25,38,52].

Phenotypic analyses revealed that co–infection with VdPV1 and VdMoV1 significantly suppresses vegetative growth and conidiation in *V. dahliae*, while exerting limited influence on microsclerotial development. This differential effect suggests that viral regulation may preferentially target pathways associated with active growth and asexual reproduction rather than survival structures. From the virus’s perspective, excessive impairment of microsclerotium formation would jeopardize the host’s ecological longevity, thereby reducing the probability of viral transmission across seasons or host–free intervals. The research led by Gao, et al. [53] discovered that infection by the mycovirus VdOMV2 in *Verticillium dahliae* primarily impairs conidiation and vegetative growth of the pathogenic fungus, while exerting promotive effects on microsclerotia formation. Collectively, the above observations hint at a possible evolutionary trade–off between host fungal survival and the long–term persistence of the mycovirus, though further evidence is required to confirm this inference.

Pathogenicity assays demonstrated viral infection status corresponds to reduced pathogenicity, while virus elimination restores virulence. Importantly, co–infection resulted in a stronger hypovirulent phenotype than single–virus infection, indicating a synergistic interaction between VdPV1 and VdMoV1. Such synergistic hypovirulence has been documented in other fungal systems. For example, in *Valsa mali*, co–infection by two viruses exhibited synergistic hypovirulence; co–infection by Cryphonectria hypovirus 1 (CHV1) and Mycoreovirus 1 (MyRV1) resulted in a stronger hypovirulence effect than single infection [54,55]. Other cases, including *Botryosphaeria dothidea* and *Ustilaginoidea virens*, where multiple mycoviruses collectively exert stronger effects than individual infections [56,57]. These results suggest that viral co–infection may enhance host attenuation through additive or synergistic disruption of fungal regulatory networks. At the molecular level, the mechanisms underlying this synergistic hypovirulence remain unclear. It is plausible that VdPV1 and VdMoV1 may jointly interfere with key signaling pathways involved in fungal growth, development, and pathogenicity, or that one virus may enhance the replication or stability of the other. Future transcriptomic, proteomic, and metabolomic analyses will be necessary to elucidate the regulatory networks underlying these interactions.

Distinct cultivars of a given host plant carry divergent genetic backgrounds and display variable levels of susceptibility to phytopathogenic fungi, which can substantially modify the biological interaction between plant hosts and hypovirulent fungal strains bearing mycoviruses [58]. Only one susceptible cotton cultivar was adopted for pathogenicity testing in the present study. Follow–up research will introduce a series of cotton cultivars with differential verticillium wilt resistance to conduct repeated pathogenicity tests, so as to verify whether the viral hypovirulence effect is universally applicable across diverse cotton genetic backgrounds, and further assess the practical application value of these mycoviruses as biocontrol agents in field cotton production.

From an applied perspective, the discovery of stable vertical transmission, efficient horizontal spread, and strong synergistic hypovirulence suggests that VdPV1 and VdMoV1 represent promising candidates for biological control of cotton Verticillium wilt. However, the ecological stability of these viruses under field conditions remains uncertain. Environmental factors such as soil microbiota, temperature fluctuations, and host plant variability may influence viral transmission dynamics and hypovirulence expression [35]. Therefore, further field–based studies are required to evaluate their long–term efficacy and biosafety.

In conclusion, this study provides new insights into the transmission biology and functional interactions of two co–infecting mycoviruses in *V. dahliae*. The asymmetric transmission pattern, synergistic hypovirulence, and stable vertical inheritance collectively suggest a complex virus–virus–host interaction network. These findings not only expand the current understanding of mycovirus ecology but also offer a theoretical basis for developing mycovirus–based strategies for sustainable disease management.

## 5. Conclusions

In summary, this study identified a *V. dahliae* strain co–infected with VdPV1 and VdMoV1. Using co–infected strains as research materials, the vertical and horizontal transmission rules of the two viruses under co–infection, as well as their hypovirulent regulation on the fungal host, were revealed. This study verified the hypovirulent properties of both mycoviruses, providing valuable resources and theoretical support for the biological control of cotton Verticillium wilt utilizing hypovirulent mycoviruses.

## Data Availability

Data is contained within the article or Supplementary Materials.

## Supplementary Materials

The following supporting information can be downloaded at https://www.mdpi.com/article/10.3390/v18070795/s1. Figure S1: Sequence alignment of contigs 4838, 4530, and 5990; Figure S2: Sequence alignment of contigs 5676 and 4525; Figure S3: RT-PCR detection of fungal viruses in *Verticillium dahliae*; Table S1: Primers for amplification of *Verticillium dahliae* mycoviruses; Table S2: Fungal virus sequence statistics.

## Appendix A. Detailed Information of 21 Virus–Related Contigs

>contig–100_4838

TTACAATCATCTCAGATTGGTTGGTTGGGGCAGTTTCCACAACAACCAGCTTCTTCAGAGAAGTGTTCGAGATTCCAGTAAATCTTCTGCAGCTGAGGGTCTCTTCTTGAGAATCCAGATAAGCGGCTGATGACTTCGGTCTTCGAAGGAAAATGATCTAGTTCAATGAATCCTAATCTAACATTAGGATCGAATAAACCATGGAGTCCGGTTGGGCTGGGGTTGAAACCAGCGTATTTAAGCTCCGAGTAAATGTGTTCACAGATCGGGCGTATCTTTGGGCTACCGCAGCTAGCGTAGTAGATTCCAATGGCACGAGCCATGAGTCTTGGGTATGTATCACGGAGTGACTTCGGGTGGAGGAGTTGAGCAAGTAGCTGTTCAGGATCTCGTTTAGGATATCCATTCCAGTTAGTGTAGCTTAGAACCTGGGCACCGTGTATACCTGATGATGAGCCACATTTCTGAGGGTTTAAGCGAGCATTGAAGCGTCGTTTGGCTTCTTCAGAGAAGGCTTCGAGGAAGTCGGCCCATTGCGAAATGGGTACTAGCTTTAACGTTCCGAATATTACGTCGTCGCCGAGCACTTTGATAAAGTGATCTTCAACAACATTCAGTCCGAGTGCTTTAAGGACAGAGATTACCATTACACTATTGTAAAAGGATCCCCAAAATTGGGTACAAAATACTCCAGATGGGATTCCAGCAAACGTTCTACGGTAAACATTACCAGTAGTAGTTGTACAAGGTGTTTGTTTGTAAGCATGACCAATCCAGTTCCACAGATTTTCAAGTCTGAGAGGGTTAGTGCAAGCTTTAGGGTAAGATTCTGTTGGGCAGTAATTGCCACAAAAGCAGAAGTATGATTTGACAGCTGATCTACAGTCGTCCCACATTGAATAGTAAACTCGCATATCGAATTCACTCCAATCAAGGTTGAATATAGTCGTAAAGCTGTGCCACTGAGAGTAGAATTCATCGTTTAAGCGATACCATCCACCGTTCAATGTTTCATAATTCCAAAGCAAAGGTGTTTTAGCGTCGGTGAAGTAATTGCTAAATAAAGGCCAGAAGAACATAGCTTCAGCAAATATTAGCGGTTTAGGGACACCAAAAACAGTTCTGACTTTAGGAGGTTCGCCAGTGCGAACCAAAGCAGGTTTTACATGTAAGTCAATATGGTGTAGCTTAACATCAATTCCGTTTTTCACATTGTGAATATATTGTCTAGAATACCAAAATATTGGATTATAGCAATTGTGGAATGACATCCGAGCGTTGTCAATAACGCCTTCGCGTTTAGCGCGCTGCACCTGTAATTTGAGTTCATTGTCGTGCGTGAACGGGCGTTCAGCACTGGTTGATAGTTTCCATGGGTACCATCTTAGGTCAGTAAAATGGACGGGGTGGATAGGGTGACTTGGTTTGAACCAGAATGTGACTCTATCAATAGCCCAGTAATAGATCTCGTCTTTGATAATATCGAAAGGCTCGACATCATAGCGGAGGAAAAACTGTTCTGCAGTCTGTTCATCAGCGTGGGATCTGTAGTATCCGTTAAGGACATCATATGCCGTGGCTAAGCCAATGGTATATGCATGGTCTGTTATCGTTTTTCGAACAAGATTCTGACATTCGTAGAACCAGTCGTTAGGTAGTTTTGCGAATCTTGGAATCGACGGCCAGTGATGGTCGTGTCCGAGGAACTCTAAATTTGACATTTCTTGGTTTTCATAAAGAAAAGAATTTAAAAGATGGGAAAAAGGTGATAAAAC

>contig–100_4530

CCATCTTTTAAATTCTTTTCTTTATGAAAACCAAGAAATGTCAAATTTAGAGTTCCTCGGACACGACCATCACTGGCCGTCGATTCCAAGATTCGCAAAACTACCTAACGACTGGTTCTACGAATGTCAGAATCTTGTTCGAAAAACGATAACAGACCATGCATATACCATTGGCTTAGCCACGGCATATGATGTCCTCAACGGATACTACAGATCCCACGCTGATGAACAGACTGCAGAACAGTTTTTCCTCCGCTATGATGTCGAGCCTTTCGATATTATCAAAGACGAGATCTATTACTGGGCTATTGATAGAGTCACATTCTGGTTCAAACCAAGTCACCCTATCCACCCCGTCCATTTTACTGACCTAAGATGGTACCCATGGAAACTATCAACTAGTGCTGAACGCCCGTTCACGCACGACAATGAACTCAAATTACAGGTGCAGCGCGCTAAACGCGAAGGCGTTATTGACAACGCTCGGATGTCATTCCACAATTGCTATAATCCAATATTTTGGTATTCTAGACAATATATTCACAATGTGAAAAACGGAACTGATGTTAAGCTACACCATATTGACTTACATGTAAAACCTGCTTTGGTTCGCACTGGCGAACCTCCTAAAGTCAGAACTGTTTTTGGTGTCCCTAAACCGCTAATATTTGCTGAAGCTATGTTCTTCTGGCCTTTATTTAGCAATTACTTCACCGACGCTAAAACACCTTTGCTTTGGAATTATGAAACATTGAACGGTGGATGGTATCGCTTAAACGATGAATTCTACTCTCAGTGGCACAGCTTTACGACTATATTCAACCTTGATTGGAGTGAATTCGATATGCGAGTTTACTATTCAATGTGGGACGACTGTAGATCAGCTGTCAAATCATACTTCTGCTTTTGTGGCAATTACTGCCCAACAGAATCTTACCCTAAAGCTTGCACTAACCCTCTCAGACTTGAAAATCTGTGGAACTGGATTGGTCACGCTTACAAACAAACACCTTGTACAACTACTACTGGTAATGTTTACCGTAGAACGTTTGCTGGAATCCCATCTGGAGTATTTTGTACCCAATTTTGGGGATCCTTTTACAATAGTGTAATGGTAATCTCTGTCCTTAAAGCACTCGGACTGAATGTTGTTGAAGATCACTTTATCAAAGTGCTCGGCGACGACGTAATATTCGGAACGTTAAAGCTAGTACCCATTTCGCAATGGGCCGACTTCCTCGAAGCCTTCTCTGAAGAAGCCAAACGACGCTTCAATGCTCGCTTAAACCCTCAGAAATGTGGCTCATCATCAGGTATACACGGTGCCCAGGTTCTAAGCTACACTAACTGGAATGGATATCCTAAACGAGATCCTGAACAGCTACTTGCTCAACTCCTCCACCCGAAGTCACTCCGTGATACATACCCAAGACTCATGGCTCGTGCCATTGGAATCTACTACGCTAGCTGCGGTAGCCCAAAGATACGCCCGATCTGTGAACACATTTACTCGGAGCTTAAATACGCTGGTTTCAACCCCAGCCCAACCGGACTCCATGGTTTATTCGATCCTAATGTTAGATTAGGATTCATTGAACTAGATCATTTTCCTTCGAAGACCGAAGTCATCAGCCGCTTATCTGGATTCTCAAGAAGAGACCCTCAGCTGCAGAAGATTTACTGGAATCTCGAACACTTCTCTGAAGAAGCTGGTTGTTGTGGAAACTGCCCCAACCAACCAATCTGAGACGATTGTAACCCTTACTAATTTATGTACTAAGCACCATTAGAGTGCAATTTTGTACTATTACTAATTCTCTA

>contig–100_5990

TTTTTCCCATCTTTTAAATTCTTTTCTTTATGAAAACCAAGAAATGTCAAATTTAGAGTTCCTCGGACACGACCATCACTGGCCGTCGATTCCAAGATTCGCAAAACTACCTAACGACTGGTTCTACGAATGTCAGAATCTTGTTCGAAAAACGATAACAGACCATGCATATACCATTGGCTTAGCCACGGCATATGATGTCCTTAACGGATACTACAGATCCCACGCTGATGAACAGACTGCAGAACAGTTTTTCCTCCGCTATGATGTCGAGCCTTTCGATATTATCAAAGACGAGATCTATTACTGGGCTATTGATAGAGTCACATTCTGGTTCAAACCAAGTCACCCTATCCACCCCGTCCATTTTACTGACCTAAGATGGTACCCATGGAAACTATCAACCAGTGCTGAACGCCCGTTCACGCACGACAATGAACTCAAATTACAGGTGCAGCGCGCTAAACGCGAAGGCGTTATTGACAACGCTCGGATGTCATTCCACAATTGCTATAATCCAATATTTTGGTATTCTAGACAATATATTCACAATGTGAAAAACGGAATTGATGTTAAGCTACACCATATTGACTTACATGTAAAACCTGCTTTGGTTCGCACTGGCGAACCTCCTAAAGTCAGAACTGTTTTTGGTGTCCCTAAACCGCTAATATTTGCTGAAGCTATGTTCTTCTGGCCTTTATTTAGCAATTACTTCACCGACGCTAAAACACCTTTGCTTTGGAATTATGAAACATTGAACGGTGGATGGTATCGCTTAAACGATGAATTCTACTCTCAGTGGCACAGCTTTACGACTATATTCAACCTTGATTGGAGTGAATTCGATATGCGAGTTTACTATTCAATGTGGGACGACTGTAGATCAGCTGTCAAATCATACTTCTGCTTTTGTGGCAATTACTGCCCAACAGAATCTTACCCTAAAGCTTGCACTAACCCTCTCAGACTTGAAAATCTGTGGAACTGGATTGGTCATGCTTACAAACAAACACCTTGTACAACTACTACTGGTAATGTTTACCGTAGAACGTTTGCTGGAATCCCATCTGGAGTATTTTGTACCCAATTTTGGGGATCCTTTTACAATAGTGTAATGGTAATCTCTGTCCTTAAAGCACTCGGACTGAATGTTGTTGAAGATCACTTTATCAAAGTGCTCGGCGACGACGTAATATTCGGAACGTTAAAGCTAGTACCCATTTCGCAATGGGCCGACTTCCTCGAAGCCTTCTCTGAAGAAGCCAAACGACGCTTCAATGCTCGCTTAAACCCTCAGAAATGTGGCTCATCATCAGGTATACACGGTGCCCAGGTTCTAAGCTACACTAACTGGAATGGATATCCTAAACGAGATCCTGAACAGCTACTTGCTCAACTCCTCCACCCGAAGTCACTCCGTGATACATACCCAAGACTCATGGCTCGTGCCATTGGAATCTACTACGCTAGCTGCGGTAGCCCAAAGATACGCCCGATCTGTGAACACATTTACTCGGAGCTTAAATACGCTGGTTTCAACCCCAGCCCAACCGGACTCCATGGTTTATTCGA

>contig–100_5676

TTAGAGAAAAACTTACTTTTGGTCTTATATATTTAATGAATTTTAGTCCCTGTAGGTAATTTAACTACATTAACACTTATTATGAAGAATATCGAGATTATTGGCTGCAAGCCTTCGCTTGCTAACCCTATCCGTGGTAATATCGACCCCGAGTCGAACGTACACTACGGTAACATCGTCGATTATGCTTTAAGAAGATATTTGAACGCTGACGAGTTCGACAAAGTTGTCAACGGCTACCGCCGATCCCAATGGGATCCTAATGGTTTAGAAGCTGACCTGATCAAACTTGATTCAGATTATTTTGATGTTGTCAAAGATGCTCATTATTATCGAGCAATTGAACACACCAGAAAATTGTTCAAACCTGATACTCCCCTGAAGCCAATTCATTTCTCTGATTTACGTCACTATCCATGGCAGTTGTCAACTAGCATTGGTGCTCCCTTTGCTACTAGTGATGACTGGAAAGATTATGTCAATGAGAAGTATGAAAGTAATTTTACTAAATCATACTATCGCGATTTATTTCGTGAAGCTCACGGCATTAGTTTACTCCCCACTATGGTTGATCGTCGCATGACGAAACGTAACCTTTACAACGAAATGTTCTTCATTAATCGAAAGAATATACATCTAATAAAAGATGGTCACAAATCAAATGAAAATGGTCATGATCTTAAATATTGGAATACTGCTTTTGCTAGACAGCATTTGGTAGAATCCCATGAAGAAGATAAAATCCGACTAGTTTTCGGTGCTCCATCTACATTTTTGATGGCTGAACTTACTTTTATCTGGCCTTTACAGATTAGCCTATTAGCTAGAGGAGAAAACTCTCCAATGCTATGGGGTTATGAAACCACTACTGGTGGTTGGTCCAGACTGTACAAATGGGCGAACAAAACTGTTCCCCAATGTAATTTTGTAGCTACTTTAGACTGGAAACGGTTTGATAGAGAAGCTAGACATACTGTAATTTCTGACATTCATCAGTTGGTCATGAGAACTTATTTTAACTTTTCTGATGGCTATCATCCCACGGTCCATTACCCCGATTCAACTGGAGCTAATCCAAGAAGAATTGAAAATTTATGGAATTGGATGACTGATGCAACACTAACTACACCACTAATGTTACCTAACGGTGATATATTAAGATTTAGACATTCTGGTATTTATTCTGGATATTTCCAGACACAGATATTAGATTCAATGTACAATTGTGTCATGATCTTTACCATCTTATCCAAGATGGGCTTCGATCTTGAGAGAAT

>contig–100_4525

GGGACGCCGATAAAGTGTGTTTGTGGCCAGTGCTTCTCAGTTACTAAGTCGCGGACTGGTTCTTGCAAGAGACGAACAGTTTCAAAATAGGTTGGAAAATGAGATATATCTATCTCAGCATAACCTGGAACATAGTTCTGTCTATAGCGTAATCCACCTGGTAAACCATGGGGATCAGGGCTATATCCTCCTGCTTTCAGGAAATTATAGATGTCCTCGCAAATTTGGTATACACGAGTGTGGTTTCCACAGTTAGCATAAGCAATACCAATGGCGCGAGCCATTAAGGCCGGTAGTGAGACCGTCCTTTCGGGATGCCTTAACATAGCTAAAAGTTGAAGTTCGTCACGGTACGGCATAGTAGCGTGATTCCGATACCTCAGCACTTCAGCATTTTCGAGACTTGGTAAGATTTCACTCTTATCAATGCTCAAAGTTGAGTTAAAATAAGCTTTCGCATAATGAGCGAAAAACCGTAGAAATGAGGGCTTGAGAGTTTCATATAAATAAGCCATTAAAAGAATGGAGTCATCTCCTTGTACTTTTATAGCTATTCTCTCAAGATCGAAGCCCATCTTGGATAAGATGGTAAAGATCATGACACAATTGTACATTGAATCTAATATCTGTGTCTGGAAATATCCAGAATAAATACCAGAATGTCTAAATCTTAATATATCACCGTTAGGTAACATTAGTGGTGTAGTTAGTGTTGCATCAGTCATCCAATTCCATAAATTTTCAATTCTTCTTGGATTAGCTCCAGTTGAATCGGGGTAATGGACCGTGGGATGATAGCCATCAGAAAAGTTAAAATAAGTTCTCATGACCAACTGATGAATGTCAGAAATTACAGTATGTCTAGCTTCTCTATCAAACCGTTTCCAGTCTAAAGTAGCTACAAAATTACATTGGGGAACAGTTTTGTTCGCCCATTTGTACAGTCTGGACCAACCACCAGTAGTGGTTTCATAACCCCATAGCATTGGAGAGTTTTCTCCTCTAGCTAATAGGCTAATCTGTAAAGGCCAGATAAAAGTAAGTTCAGCCATCAAAAATGTAGATGGAGCACCGAAAACTAGTCGGATTTTATCTTCTTCATGGGATTCTACCAAATGCTGTCTAGCAAAAGCAGTATTCCAATATTTAAGATCATGACCATTTTCATTTGATTTGTGACCATCTTTTATTAGATGTATATTCTTTCGATTAATGAAGAACATTTCGTTGTAAAGGTTACGTTTCGTCATGCGACGATCAACCATAGTGGGGAGTAAACTAATGCCGTGAGCTTCACGAAATAAATCGCGATAGTATGATTTAGTAAAATTACTTTCATACTTCTCATTGACATAATCTTTCCAGTCATCACTAGTAGCAAAGGGAGCACCAATGCTAGTTGACAACTGCCATGGATAGTGACGTAAATCAGAGAAATGAATTGGCTTCAGGGGAGTATCAGGTTTGAACAATTTTCTGGTGTGTTCAATTGCTCGATAATAATGAGCATCTTTGACAACATCAAAATAATCTGAATCAAGTTTGATCAGGTCAGCTTCTAAACCATTAGGATCCCATTGGGATCGGCGGTAGCCGTTGACAACTTTGTCGAACTCGTCAGCGTTCAAATATCTTCTTAAAGCATAATCGACGATGTTACCGTAGTGTACGTTCGACTCGGGGTCGATATTACCACGGATAGGGTTAGCAAGCGAAGGCTTGCAGCCAATAATCTCGATATTCTTCATAATAAGTGTTAATGTAGTTAAATTACCTACAGGGACTAAAATTCATTAAATATATAAGACCAAAAGTAAGTTTTTC

>contig–100_19074

GTATTTACCGGGTTGGGGCTTGGGAACTGAGATGCCGGAGGAGAACGCCGATGCCTTGCTGGCACTAGCTGCCTGACCGGGTTGAGAACCAACCGCTGATCGACGAGCTTGGCGTTCAGCCTTACCAGGCTTAGACTTACGTCCAGCCGTGGAGGCGGAGTCGCTGGGGGCGACGGTAGAGGGGAGTTGCGACTGGGTGTCAGCCATGGCCGCAAAGTGAAGGAGGCAGGGGGGGGTGTTATATTCTTGGAGGCTTGAAAGGCTTAAATATAACGAGAAACACCAGATAAACTGGTGAAAAGACAAG

>contig–100_19083

GCTGGGCCAAACGCAGGAGAGCAGCGACTACGAGATCCTTGGTGAACTCATGGTCGTCTATCTCAGCATGAGACTTAAACTCTGCATACTTGGCGTTGACCGTAAACCGATCCGGAAAGGTCAGGAGCGAACGTTCGAGAACACGCTCGTCGATTGCAAAGGTCTGGTCCCGAGCCGGTTCACCCGCACCCGTCTGAAAAACGACAGAGTATTTACCGGGTTGGGGCTTGGGAACTGAGATGCCGGAGGAGAACGCCGATGCCTTGCTGGCACTAGCTGCCTGACCGGGTTGAGAACCAACCGCTGA

>contig–100_5397

GTCTAGCGCTTTTACCGTGTCAACGGGTTTATGCCTCCTTCATTCCTTGAGCGAAATGGAAGATTTTACTCAAGATCCAACAACACACAACATCGTTGCTGAGGGGTCTCATTTAATTGACGCACTCCACCTCCGGCCCCCAAAGCAGAGGTCAGCCACCAGCGAAGATGTTGTTTCTTCCAACTTCAGGTCTCCCAACCTGGCGGAAATCGCAAAATACGGGGGGTACTCAACGTACTCGTCTAACTCTAACACCGATCCTTATGTGAGAGAGACACTAAAACTATTTTCTCGAGACACCTACGAGGATATCCGTGGTTTCACTCGTCGTCCGCAAGGGACTCCAGGCATGTACACTGCCTTGAAGAAGTTCAGCGGAGAGCGAAACACATTCGGTGATCTCTCCCCTAGTCAGCAGTCTTCGATGCGCCGTGCCATCGGCAAAGCGAAGAAAGCTTTCAAACTCCCCTACAAGCGCGAGCCGCTTGATTGGCATGAAGTGGGTCAGTTTCTAAGGCGTGATACCGCCGCAGGAGCAACCTTCATGGGCCAAAAGAAGGGTGATGTGATGGAAGACATTTACCACGAAGCTAGGTGGTTAGGACACCGGATGAAACAGGATGGTAGGGCCGGTTTCGATCCAACCCGAATGAGGTTCCCTCCTTGTTTGGCAGGCCAGCGTGGGGGTATGTCGGAGATTGACGACCCCAAAACGCGCCTGGTTTGGATTTACCCTGCTGAGATGTTGGTAGTTGAAGGGTTCTACGCCCCTTTAATGTATCGTGACTTTATGATCGATCCAAATTCACCAATGTTGAACGGGAAGAGCGCGCAGCGTTTGTACACCGAATGGTGCTGCAAGCTAAGGGATGGGGAAACACTATATGGCATTGACTTTAGTGCTTTTGACACCAAAGTCCCAGCGTGGCTAATTCGCGTGGCATTCGATATTCTGCGTCAGAATATTAACTTTGAGACGTTCGGTGGTAAACCAGTCAAGAAGCAGGATGCTCAGAAGTGGCGAAACGTTTGGGATGCCATGGTGTGGTATTTCATAAATACACCCATACTTATGCCGGACGGCCGGATGTTCCGGAAGTTTCGGGGTGTACCCTCCGGATCGTGGTGGACCCAGATGATCGATTCAGTCGTAAATCATATCCTGATTGATTATCTTGCGGACTGCCAGCGTGTAGGGATCCGAAACCTGAGGGTGTTGGGAGATGATAGCGCTTTTTGCTCTGGTGACCAGTTTGATCTGGAATTAGCAAAAGGTGACTGCGAGAACACCGGTATGGTGATAAAGCCCGAGAAGTGTGAACGAACCAAAGACCCAGGTGAGTTCAAGCTATTAGGCACGACGTACCGGGGGGGCCACGTTTTCCGTGATACGGAGGAGTGGTTCAAACTCGCTCTCTATCCCGAGTCCAGCGTGCTGACGTTGGATATTTCTTTCACCAGGTTGATTGGCCTGTGGCTCGGTGGAGCAATGTGGGATAAGGAGTTCTGTGAATACATGGACTTCTTCCAATCAAGCTATCCTTGTCCTGAGGAGGGGTGGTTCTCCAAAGATCAGAAAAGGTGGCTTGAGATAATCTACTCAGGCAAAGCGCCAAGAGGTTGGACTTCCAAGAAGAGTCTCTTTTGGCGATCAATCTTCTATGCCTACGGCTAGGAGTGACAAATCTTCTGCAGATAGCAGAGTCTTGCGTGTATACGCGGATGTGACATTGACACGCCGGGGCGACGGATGGTGATCCCCCTGGCCAGTGCACGTCTGCTGTCAGATAAAGTCTCCCGTGAACTTTACCCGGTGGTGCATATCGG

>contig–100_7674

CCCAAGGCGATTTCGCCCCCGTTGCTTCAAGCGACGTCCGTGTTCCCGCTTCGCTAGCTGCGTTTATTTCGCAGTTTGGTGAGTTCTCCGTTCCCACTTTAGGGACTCGGTTTTTATTCTCGGATTACGAGAATACCGTACGTTCTATCGTATGGATGGCCGAAGGAGTAAAGCGCAACGGAATTGGAGGCGCTCCTTTGAAGAGATCGTGGTTGCCCGTCCGTCCATCTGACGGGCACACAAAGACCGTGATAGCTTCACGCCTGTGCGAGCTTCTGGCGCAAGCAGAGCTCGAAGTCAATCCTGACGTCCTTGAGGGGGGCGTACTTTCAGGAGAGATTCCCGATGCTTGGGAGAGCATCAAACCCGTCCTTGGAGACGGTGACGAACGTCGAGACAGATTCGACTTTTTATTTAAGTCGTATCGTGATGCTCCTGCGTTCGTCACTGCCTTCACTACCACCAGTGCATCTGGTGTGTTGGCTGAGTTAGATCTTAGCTGGGACCGTCCGTCCGCGGGTCACGTAGACTGGACTTTTAACCCGAAAGAAGTGTTTACTCGCCTTTCGGATGCCTGGGCACGTAGGTCCACAACCTACGCCCTATTCTTCGAGTTGTCGTCGAGTCAGATGACGCGTAACGTGTCAGCTGGGAGCCAGTCCCAGATGGCGCGTGTGAACACCCGTGATGGTGTGACCGTCGTCAAGACTCATCTTGGTTTGTCGGCCCCCGAGTTCTCACTCGTGGCATGCTTCCCCGCATCAGTTATCTTTACTGGTGGGCTGACCCGTCGCGTGGTGGTGACCACGCCACTCTCTGTTGAACAGAGAGCCACTGAATTCGTTCAGATGGATTGGCGCTAGTCACTGCGGTAACGCAAAAAGACAACCTACCCAACTCAGTAGGAAAGGCAGTCGTGTAAACGACACCGTGACTCCGTGTATCGGAGAAGTCCTGTGTATCAGGCACCCAACTTTTTAACAAGAAGTGGGAAACGTGACAGTTTATCTGTCGTGTGTCCGTAAAACTCCCCCGAGTTCTCACTCGTGGCATGCTTC

>contig–100_15097

TCTATCTTTCGCTTTCAAAAGAAGAACCCCTCCCCTCACTTCCACTGGAATATGCGCATACCCCCTTCCCTCAGGGGTTTGCGTCTTATACTCGGGTGGCAGCGGGTCTTTCGAAGGGGGCCTGGCGTCGGGCCCGGGTCGTTGAGGGTTCCTTCCACCTTGCGTGCAGTTTGTATGCCCAGATGCCCGTCCGCCCCCCTTCTTGCGGGGACTGGCGGGCGGTCCTCCAGCGCGGCACGCCTCGCTTCTGGGAGCCCAGATCTTGTATCCGCAAGTTGTTCGAACAAATTTGGCGGAGACTCTGTGGCCCTTTTGCGCAGGTGGTGGGGGCAGGAAGGACGGAGGAGAGGCTGATGTTGCCGGTCGGGGAGGAAGAAGAGGTACAAAG

>contig–100_15987

GAGTCTTGCCCTTGGACAAGACTAGGCCGGAACCCACAACCCCATCCGCCCACTTCTCTGCCTCCTCAGGACGACAACGGAACACAATGTCGTCCCCGTGCACGCGGACTGGAACTCCCCTACGGGGAACCAGCCAGCGGAAGACGAGGTAATTAACGAGACAGAGAAGAGGGAAGGATATGAAGGTCCCCATCAGCTGACCGCGTCGCTGCGGTGCGCACAGTTTACTTGTGTACAAGAAGGCTTGCGACCTTGCCAAGGCAAGGGCCCAAACCGACTTAGGTACACAGGGAGAGAGGCTCGAGAGGACTCGCAAGACGCACTGACTAACTTCCATCGATAAGTTATCAGTAGCCGCCTCGTAGT

>contig–100_17699

GGCCGGAACCCACAACCCCATCCGCCCACTTCTCTGCCTCCTCAGGACGACAACGGAACACAATGTCGTCCCCGTTCACGCGGACTGGAACTCCCCTACTGGGAACCAGCCAGCGGAAGACGAGGTAATTAACGAGACAGAGAAGAGGGAAGGATAGGAAGGTCCCCATCAGCGGACCGCGTCGCTGCGGTGCGCACAGTTTACTTGTGTACAAGAAGGCTTGCGACCTGGCCAAGGCAAGGTCCCAAACCGACTTAGGTACACAGGGAGAGAGGCTCGAGAGGACTCGCAAGACGCACTGACTAACTTCCATCGATAAGTTATCAGTA

>contig–100_19940

GAAAAAGGAAATACCGCGCTTGTTCCCCCGCGGTTGGGATCGCCACTATCGCTCCAGAGCGGGTGGTCTTCTTCTTCCTGTGAAGTCCTGCCTTGAGGCAGGTCGTAGGAGAGGTGGAGGTCGTTACCGGAATTCGCAGCTCCGGTACCTCCGTCGTGTTGCGCTAGGCTGGGAGACTGTGGAGTCTCCCGACGCCCGGGCGCGGGTGACATGTGTTCCGGACGGGTGTAAACTCCGTACGGTGACTGTGTCATCCGTGGACCAGGCGGCTCTTAAGCCCTTGCACGAGACCCT

>contig–100_20798

ATCAGCCTCTCCTCCGTCCTTCCTGCCCCCACCACCTGCGCAAAAGGGCCACAGAGTCTCCGCCAAGTTTGTTCGAACAACTTGCGGATACAAGATCTGTGCTCCCAGAAGCGAGGCGTGCCGCGCCGGAGAACCGCCCGCCAGTCCCCGCAAGAAGGGGGGCGGACGGGCATCTGGGCATGCAAACGGCACGCAAGGTGGAAGGAACCCTCAACGACCCGGGCCCTACGCCAGGCCCCCTTCGAAAGACCCGCTTCCACCCGAGTATAAGACGCAAACCC

>contig–100_20854

AAGGCTTGCGACCTTGCCAAGGCAAGGTCCCAAACCGACTTAGGTACACAGGGAGAGAGGCTCGAGAGGACTCGCAAGACGCACTGACTAACTTCCATCAATAAGTTATCAGTAGCCGCCTCGTAGTCCCCGGAGACATAAACCTCCCCTTTTCGCAGGCCGAAAGAAGTAAACTGCCCGTGCACAGCGTCACCGCGGAGTAGCCAGTCCTGAGTTGAGATGTGGTCATAGAGGGTCTCGTGCAAGGGCTTAAGAGCCGCCTGGTCCACGGATGACACAG

>contig–100_29403

GGCGGCTACTGATAACTTATCGATGGAAGTTAGTCAGTGCGTCTTGCGAGTCCTCTCGAGCCTCTCTCCCTGTGTACCCAAGTCGGTTTGGGACCTTGCCTTGGCAAGGTCGCAAGCCTTCTTGTACACAAGTAAACTGTGCGCACCGCAGCGACGCGGTCAGCTGATGGGGACCTTACTATCCTTCCCTCTTCTCTGTCT

>contig–100_11639

GGGTGCCAACCTGCAGATTCCTCTTATGTGGCAGCCCGTTTATGATCGTTTGGTTGGTCTGGCGCCGAACGTCGCAGCCATGTCTTCTGGTGTCACTGGTGCTCTTCGCGCTCTGCCCCCCTTCGTGCAGTTTCCGAACATCCAGGTCCGCCCTGATGTTTTGCAGTACCAGTTCACGGCTGCTGATCGCGTTCTTGCCCGCCACCTGGCTTCCGACCTTAAGCAGTATCCCCAGGTTCTCAACTACCGCGAGGTCGGTGGTCCCTCTGTCGACAACCTCACTCACTCGTTGGCTCAGGAGGTGAAGTACTCCCCTCTCCCCTCCGTTGAGGTCGTCCTGCTCAACGGTGCCCCGGGTTGCGGTAAGAGTTTTTGGCTCCGTAACGCGGTCCGTGCGTTTGAGGCTGATGGCCCGGTGCGCTTTCACACCTGGAACAATCGCCTTCGTGTCGACATTGAGCGTACCTTTGAGCCTATTCTGTCGTATTGGGACGATCGCTCCGCTTGTTCTGGCATGACCCCTCTTTTCCAGAAG

>contig–100_9446

CCGGGTTGCGCCGGGGGGAAGGGGGCTTGTTCCCATCACAGTGTGTTTTTCTCACTGCTTCACGGAGCGCGGTACCCCCACCTTCACCGCCGTTGAAGGATCGGGACACTAGCACGATGGAGTGCACCAGCCCACTTTAACTGGCCACCGTACCTAATAACAGGGTGTCACCCTGGACCTTCGTGGGTTTTAGGAGAACAAGAGAGCGGAGGTTACAACCAGCAGCCAGGCTGGAACCGGGTACGACACCGGTAGCGGTCTTAAGGGGAACCGAACCCACGGACGCCACGCAAGGTGGCAAAAGAGGGAGAGGAAGGGACGGGAGAACAAAGACAGAGAGGGCAACGAAACGGAAGAAGAAGGAGGGGAGCTAAACAACACGGGGGGGATAACGGGAGGGAGGAAGGTGGAAAAGCTCACGGGCTGACAAAGAGACGGCGACGGCAGACGAGAGGACGAGGGAAGGAGTGAAAACATCGTCTGAGGCAAAACGCAATGACAAATCGAAAGAATCCCAGAAGTCGGCGTCGGAGCGACCATCTTCAAGCGCGAGGGTACCCCGGTGCAGCAAAACGTCGGGAGAGATGTGAAGAGAGTGAAACCCGTAAACAAAGCCACAAAACTCACCCACATCACCCACGCCCAGCTTCGGAGAGAAAGGGTACGAGTCGGAGAGGACATGATCAAAGCGGCCGCAGAGGATCATGTCGTCACCAGAAAAGCAGGCGGGGACGTCGG

>contig–100_10957

CTAACCGTGAGAAGCCCGCTTTCCAGGGTCGCCTTCTCAATGTCCAGCACGCCTTGCGCCACCAGCTGCCTGTCCCCGCTGATACTGTCCTTGTTCGCCCTCGTCTTCCCGCGGTAGTCCCGGATGCTGTCCCTGTTGGTCTCCCCGACCCTGTTGTCGGCGCGCACCCTGACCTCCTCCCTGAGTCTCGTGAGTGGTTCGTGTCCTCGGTCGGCTACACGCAGCAGGTGTCGCTCAATGGCTCCCCTCTCGGCCTGCATCATACCAGGCGCGATGCTGCTACTGAGCAACTCTCTTTGGCGGAGCGGATTCGGCCTCAGGTCCCGCTGGTTCGTGCGCGTCATCGTCGTCAGGCTGCTGACTTGCGTCGTGGACTCTCCTCCTTTTTGTCGCTTGGTGGCTACGCCTTCAAGTTCGGTCTTTACCAGGATTGCCTGGTTGAGCAGCTTGAGTCCTGGGTTTCCGGTCGTACCCGTGCTGACATTGAACGGGCAGTGGCCAACTCCCCCCCTGACTGGGATCGTCTGTTCGTCCGCCTGTTCCTGAAGTCCCAGCGCGTTAAGAAGATTGCCAAGCGTCATGCGCCTGCTGGTAAGGGCCAGATCGTCACCGATGTCTCCCATGTTAAGCTGTTCGGTGATGCTTTCTGGGCCCTGTACCTCGAGCGGTCTGTGCTGCGTTCCCTTCGCCCCGGTTTCTGTCTGCACACTCGCTCCAATGTCACCGCTATGCGCTCCTG

>contig–100_6649

CGAGCGGTCTGTGCTGCGTTCCCTTCGCCCCGGTGTCTATCTGCACACTCGCTCCAGTGTCACCGCTATGCGCTCCTGGTACTCCCGTTGGTGGCGTCCTGGTTCTGGCGTCACCTGGTGCGACTACACCGGGTGGGATTCCGGGGTGAACGAGTCTTTCACCTTGCTCTACACTGATGTCCTCCGCTCGTACGGTGTCCCCTCCGCCGTGGCTGCGCGTTTCGCCTCTGACCGCCATTCGCTCCGTTCCTTTCTGGGCCCGATGCCCGCCATGCAGGCGTCTGGTGATCGTTACACTTGGCTTGCGAACACCCTCGGTAATATGGCGCTCACTGGGATCTCGTTTGACTTGCCCCCCGACGTCCCCGCCTGCTTTTCTGGTGACGACATGATCCTCTGCGGCCGCTTTGATCATGTCCTCTCCGACTCGTACCCTTTCTCTCCGAAGCTGGGCGTGGGTGATGTGGGTGAGTTTTGTGGCTTTGTTTACGGGTCCCACTCTCTTCATATCTCTCCCGACGTTTTGCTGCACCGGGGTACCCTCGCGCTTGAAGATGGTCGCTCCGACGCCGACTTCTGGGATTCTTTCGATTTGTCATTGCGTTTTGCCTCAGACGATGTTTTCACTCCTTCCCTCGTCCTCTCGTCTGCCGTCGCCGTCTCTTTGTCAGCCCGTGAGCTTTTCCACCTTCCTCCCTCCCGTTATCCCCCCCGTGTTGTTTAGCTCCCCTCCTTCTTCTTCCGTTTCGTTGCCCTCTCTGTCTTTGTTCTCCCGTCCCTTCCTCTCCCTCTTTTGCCACCTTGCGTGGCGTCCGTGGGTTCGGTTCCCCTTAAGACCGCTACCGGTGTCGTACCCGGTTCCAGCCTGGCTGCTGGTTGTAACCTCCGCTCTCTTGTTCTCCTAAAACCCACGAAGGTCCAGGGTGACACCCTGTTATTAGGTACGGTGGCCAGTTAAAGTGGGCTGGTGCACTCCATCGTGCTAGTGTCCCGATCCTTCAACGGCGGTGAAGGTGGGGGTACCGCGCTCCGTGAAGCAGTGAGAAAAACACACTGTGATGGTAACAATCCCCCTTCCCCCCGGCGCAACCTGGGCCGCATTAGAAGGAGCCGAAATCCGTTAATTCCGTCCCATTCTCGATCATGGGATGTCACGCTGCGGAGCGTGGCTGTGCAGAGGCCGTACGGACACCGGCACTTCCTCGCGCTGTAAGACACGGTCCACAACACCTCATTCAACTGAGGCTTCAAGATCGCTTCCCTTTCTCACACTTTTCCTTGTTTGCGTTTGTTTGTTGCGTTTCCTCATCCTCTCTGTTCCCTTTCTATTGTTATGTTGTCTCCCACCGCCTCCCTTGGGATCGCTCTCCTTGATGTTGATTTGGTTGATCTTGATCGGGCTCGCCTCCTCTGTGAGCTTGATCTTCGCGTGGCTCAGGCCGCGTTGTCCGAGGTTCGTGCTCGTTTCCCCCCCCCCCCCTCCCCCCCCCCCCCCCCCCCCCCCCCCCCCCCCCCCCCCCCCCCCCCCCCCCCCCCC

>contig–100_6965

CATCAATCGAAAGACCCTGAGCGTCGACAAGGGGGTACGTGGGGGTGCCACCCTTGGACTTAGACTCGGCGAACCGGGGAGAGGTGACGAGCAGCGGGACGTCCCGAGGCGGATTGGAAACCACGACAACAGCACCATGGGACACCTGAGCACCCACGTCGAGGGCGCGGGGCAAACCGAAGAGCGCAGAATTCTCAATCGACAACCGGCGATTCACACACGAGTAAGAGGTGGAAAGGGAATTCAACCAATCACAAGTGGGGGGGTCGGAACGGGAGAGAGAGTTGGCCTCAGGAAAGGGAGGGTGAGCCTGCGAAGAATCGAACGTGAAGACCATACGCCGAAGACCGGGGCTGGTGAGCGCGATCATCTGGATCATACCGGGGTAAAGAAGGGCGGCATCATCGAAGACCAAGGTACCGGAGCTCTTCTGGAAAAGAGGGGTCATGCCAGAACAAGCGGAGCGATCGTCCCAATACGACAGAATAGGCTCAAAGGTACGCTCAATGTCGACACGAAGGCGATTGTTCCAGGTGTGAAAGCGCACCGGGCCATCAGCCTCAAACGCACGGACCGCGTTACGGAGCCAAAAACTCTTACCGCAACCCGGGGCACCGTTGAGCAGGACGACCTCAACGGAGGGGAGAGGGGAGTACTTCACCTCCTGAGCCAACGAGTGAGTGAGGTTGTCGACAGAGGGACCACCGACCTCGCGGTAGTTGAGAACCTGGGGATACTGCTTAAGGTCGGAAGCCAGGTGGCGGGCAAGAACGCGATCAGCAGCCGTGAACTGGTACTGCAAAACATCAGGGCGGACCTGGATGTTCGGAAACTGCACGAAGGGGGGCAGAGCGCGAAGAGCACCAGTGACACCAGAAGACATGGCTGCGACGTTCGGCGCCAGACCAACCAAACGATCATAAACGGGCTGCCACATAAGAGGAATCTGCAGGTCGGCACCCATATCAAACCAGGAGACACGGACGGAAGAAAGGCGCACTAGGAGGTTGGGGAGCGGAGGCACCACAGCAGCGCCAAACTGGGGGAGGCCAAAACGAAGGTGAAGGTCGGCGCCAGCGCCGTAAAGGGAGGAGGAGCCAGAAGGATACGTGGCCCGAGCCGGCTGCACGAAAAGAGGGGGAGCGCCGAGCTGCGCCTGAGGCAAGCCGTTACCAAGAAGCGCGGCTGAGTAAATCGTAAGCCCGACCTGGAAATAGGCGAGGACCCGGGGCAAATCCTGCAAAGCCACAGTGCCATCATCAAACGCCGCGAAGGCAGCGGGGGGAACGGACGAAAGGAAAGCGGCCAAGCAAGTGAGGGGCGGCAAGCCAAGGAGCGCACCAAGGCAATCCCAAACACGGATCCGAGCAGGGTCAGCCCTGGGAATGAAAGGCACCGGGAGACGGAGAAGAGCGCCGCGCCAGAGGGGGAAATCAAGAGTGGCCGGG
