# Supplementary material for: Co–Infecting Mycoviruses VdPV1 and VdMoV1 Attenuate Verticillium dahliae and Are Transmitted Vertically and Horizontally
Source: Viruses. 2026 Jul 19;18(7):795. doi: 10.3390/v18070795 (PMC13431470; doi:10.3390/v18070795)
Supplement: Supplementary file 1 [file viruses-18-00795-s001.zip › viruses-4433058-supplementary.pdf]

Supplementary Materials

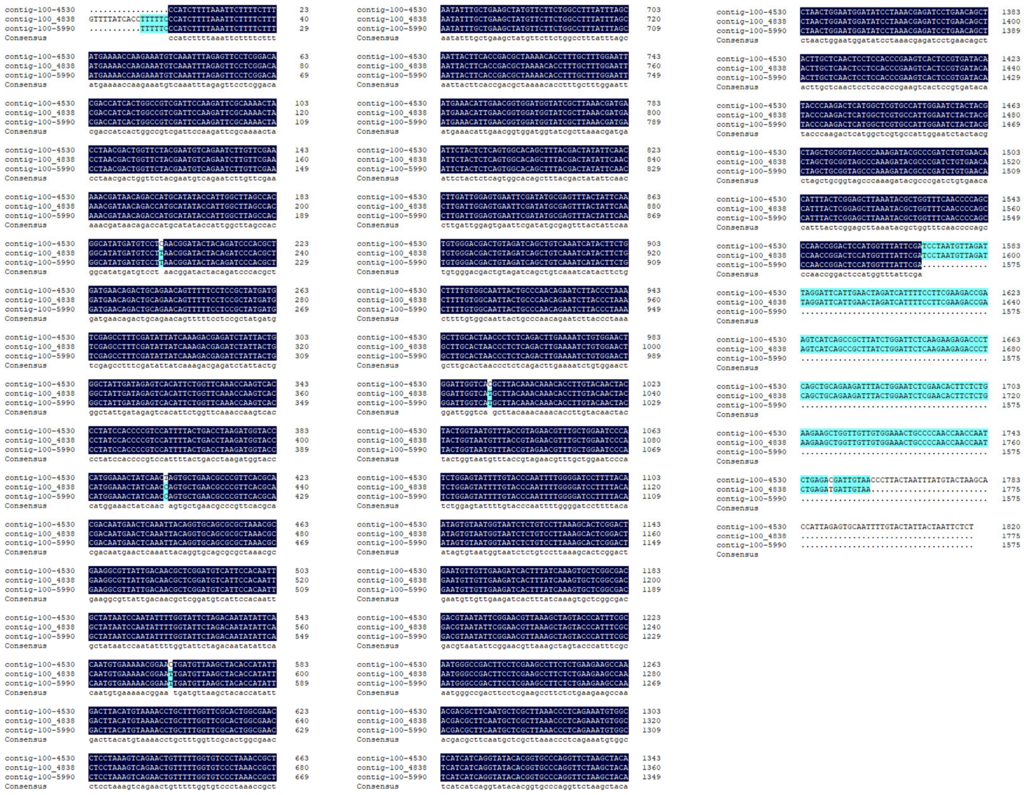

Supplementary Figure S1: Sequence alignment of contigs 4838, 4530, and 5990. Regions shaded in black represent the overlapping segments shared by all three contigs, while blue regions indicate sequences overlapping between two contigs.

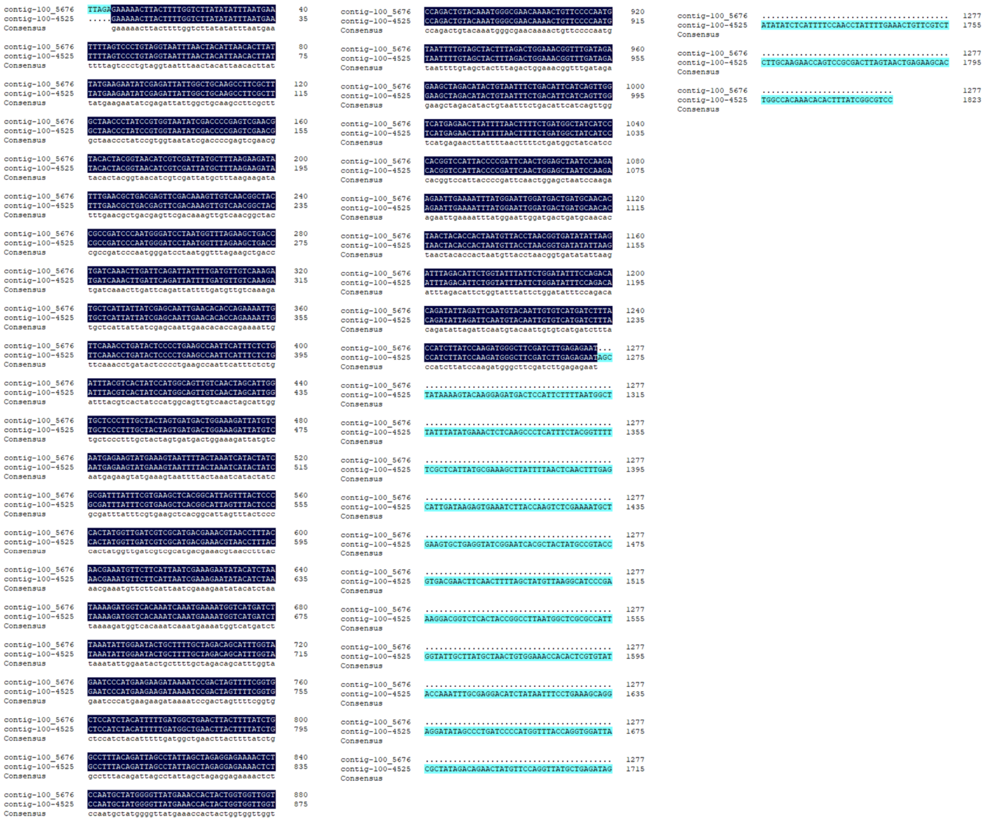

Supplementary Figure S2: Sequence alignment of contigs 5676 and 4525. Black regions represent the overlapping segments shared by the two sequences.

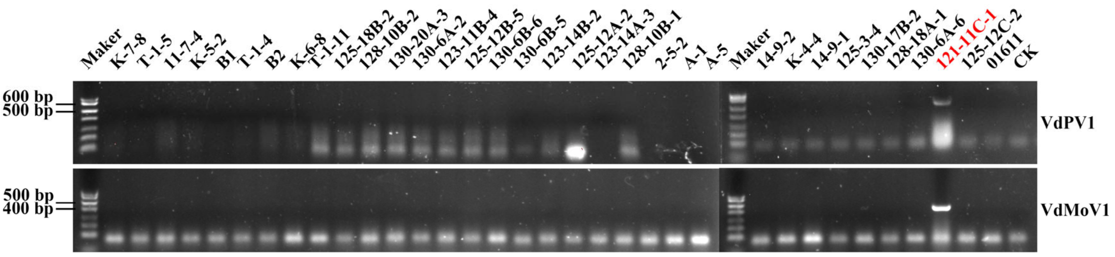

Supplementary Figure S3: RT-PCR detection of fungal viruses in *Verticillium dahliae*

Supplementary Table S1: Primers for amplification of *Verticillium dahliae* mycoviruses.

Primer	Sequence（5'-3'）
VdPV1-F	CGTCACTGCCTTCACTACC
VdPV1-R	ATACACAGGACTTCTCCGATAC
VdPV2-F	CAGAACTGTTTTTGGTGTCC
VdPV2-R	CTTGGGTATGTATCACGGAG
VdPV3-F	TACAAATGGGCGAACAAAAC
VdPV3-R	TTCGGGATGCCTTAACATAG
VdMov1-F	TGGACCAGGCGGCTCTTAA
VdMov1-R	GGACGACAACGGAACACAATG
LeDF2-F	ACGCCTTCAAGTTCGGTCTT
LeDF2-R	TTCGCAAGCCAAGTGTAACG

Supplementary Table S2: Fungal virus sequence statistics

Number	Contig	Length(bp)	number of contigs	Family	Best match	Identity(%)	Genome Type
1	contig-100_4838	1775	172	*Partitiviridae*	Raphanus sativus cryptic virus 1	75.72	dsRNA
2	contig-100_5676	1277			Partitiviridae sp.	72.05	
3	contig-100_4525	1824	380	*Partitiviridae*	Partitiviridae sp.	74.9	
4	contig-100_4530	1821			Raphanus sativus cryptic virus 1	75.98	
5	contig-100_5990	1575	64	*Partitiviridae*	Raphanus sativus cryptic virus 1	75.84	
6	contig-100_19074	307	37,499	*Partitiviridae*	Verticillium_dahliae_partitivirus_1	95.77	
7	contig-100_19083	307				95.44	
8	contig-100_5397	1827				95.49	
9	contig-100_7674	1058				94.14	
10	contig-100_15097	388	56,195	*Botourmiaviridae*	Verticillium dahliae magoulivirus 1	90%	(+)ssRNA
11	contig-100_15987	366				90.41%	
12	contig-100_17699	329				90.24%	
13	contig-100_19940	294				94.86%	
14	contig-100_20798	281				90.04%	
15	contig-100_20854	280				90.65%	
16	contig-100_29403	201				93.94%	

Supplementary Table S2 Continued: Fungal virus sequence statistics

Number	Contig	Length(bp)	number of contigs	Family	Best match	Identity(%)	Genome Type
17	contig-100_11639	535	69	*Deltaflexivirida*	Lentinula_edodes_deltaflexivirus_2	98.13%	(+)ssRNA
18	contig-100_9446	738				97.14%	
19	contig-100_10957	739	49	*Deltaflexivirida*	Lentinula_edodes_deltaflexivirus_2	96.89%	(+)ssRNA
20	contig-100_6649	1537				97.23%	
21	contig-100_6965	1448				97.44%	

Appendix 1. Detailed information of 21 virus-related contigs

>contig-100_4838

TTACAATCATCTCAGATTGGTTGGTTGGGGCAGTTTCCACAACAACCAGCT
TCTTCAGAGAAGTGTTCGAGATTCCAGTAAATCTTCTGCAGCTGAGGGTCT
CTTCTTGAGAATCCAGATAAGCGGCTGATGACTTCGGTCTTCGAAGGAAAA
TGATCTAGTTCAATGAATCCTAATCTAACATTAGGATCGAATAAACCATGGA
GTCCGGTTGGGCTGGGGTTGAAACCAGCGTATTTAAGCTCCGAGTAAATGT
GTTCACAGATCGGGCGTATCTTTGGGCTACCGCAGCTAGCGTAGTAGATTCC
AATGGCACGAGCCATGAGTCTTGGGTATGTATCACGGAGTGACTTCGGGTG
GAGGAGTTGAGCAAGTAGCTGTTCAGGATCTCGTTTAGGATATCCATTCCA
GTTAGTGTAGCTTAGAACCTGGGCACCGTGTATACCTGATGATGAGCCACAT
TTCTGAGGGTTTAAGCGAGCATTGAAGCGTCGTTTGGCTTCTTCAGAGAAG
GCTTCGAGGAAGTCGGCCCATTGCGAAATGGGTACTAGCTTTAACGTTCCG
AATATTACGTCGTCGCCGAGCACTTTGATAAAGTGATCTTCAACAACATTCA
GTCCGAGTGCTTTAAGGACAGAGATTACCATTACACTATTGTAAAAGGATCC
CCAAAATTGGGTACAAAATACTCCAGATGGGATTCCAGCAAACGTTCTACG
GTAAACATTACCAGTAGTAGTTGTACAAGGTGTTTGTTTGTAAGCATGACCA
ATCCAGTTCCACAGATTTTCAAGTCTGAGAGGGTTAGTGCAAGCTTTAGGG
TAAGATTCTGTTGGGCAGTAATTGCCACAAAAGCAGAAGTATGATTTGACA
GCTGATCTACAGTCGTCCCACATTGAATAGTAAACTCGCATATCGAATTCAC
TCCAATCAAGGTTGAATATAGTCGTAAAGCTGTGCCACTGAGAGTAGAATT
CATCGTTTAAGCGATACCATCCACCGTTCAATGTTTCATAATTCCAAAGCAA
AGGTGTTTTAGCGTCGGTGAAGTAATTGCTAAATAAAGGCCAGAAGAACAT
AGCTTCAGCAAATATTAGCGGTTTAGGGACACCAAAAACAGTTCTGACTTT
AGGAGGTTCGCCAGTGCGAACCAAAGCAGGTTTTACATGTAAGTCAATATG
GTGTAGCTTAACATCAATTCCGTTTTTCACATTGTGAATATATTGTCTAGAAT
ACCAAAATATTGGATTATAGCAATTGTGGAATGACATCCGAGCGTTGTCAAT
AACGCCTTCGCGTTTAGCGCGCTGCACCTGTAATTTGAGTTCATTGTCGTGC
GTGAACGGGCGTTCAGCACTGGTTGATAGTTTCCATGGGTACCATCTTAGG
TCAGTAAAATGGACGGGGTGGATAGGGTGACTTGGTTTGAACCAGAATGT
GACTCTATCAATAGCCCAGTAATAGATCTCGTCTTTGATAATATCGAAAGGC
TCGACATCATAGCGGAGGAAAAACTGTTCTGCAGTCTGTTCATCAGCGTGG
GATCTGTAGTATCCGTTAAGGACATCATATGCCGTGGCTAAGCCAATGGTATA
TGCATGGTCTGTTATCGTTTTTCGAACAAGATTCTGACATTCGTAGAACCAG
TCGTTAGGTAGTTTTGCGAATCTTGGAATCGACGGCCAGTGATGGTCGTGT
CCGAGGAACTCTAAATTTGACATTTCTTGGTTTTCATAAAGAAAAGAATTTA
AAAGATGGGAAAAAGGTGATAAAAC

>contig-100_4530

CCATCTTTTAAATTCTTTTCTTTATGAAAACCAAGAAATGTCAAATTTAGAG
TTCCTCGGACACGACCATCACTGGCCGTCGATTCCAAGATTCGCAAAACTA
CCTAACGACTGGTTCTACGAATGTCAGAATCTTGTTCGAAAAACGATAACA
GACCATGCATATACCATTGGCTTAGCCACGGCATATGATGTCCTCAACGGAT
ACTACAGATCCCACGCTGATGAACAGACTGCAGAACAGTTTTCCTCCGCT

ATGATGTCGAGCCTTTCGATATTATCAAAGACGAGATCTATTACTGGGCTATT
GATAGAGTCACATTCTGGTTCAAACCAAGTCACCCTATCCACCCCGTCCATT
TTACTGACCTAAGATGGTACCCATGGAAACTATCAACTAGTGCTGAACGCC
CGTTCACGCACGACAATGAACTCAAATTACAGGTGCAGCGCGCTAAACGC
GAAGGCGTTATTGACAACGCTCGGATGTCATTCCACAATTGCTATAATCCAA
TATTTTGGTATTCTAGACAATATATTCACAATGTGAAAAACGGAACTGATGTT
AAGCTACACCATATTGACTTACATGTAAAACCTGCTTTGGTTCGCACTGGCG
AACCTCCTAAAGTCAGAACTGTTTTTGGTGTCCCTAAACCGCTAATATTTGC
TGAAGCTATGTTCTTCTGGCCTTTATTTAGCAATTACTTCACCGACGCTAAA
ACACCTTTGCTTTGGAATTATGAAACATTGAACGGTGGATGGTATCGCTTAA
ACGATGAATTCTACTCTCAGTGGCACAGCTTTACGACTATATTCAACCTTGA
TTGGAGTGAATTCGATATGCGAGTTTACTATTCAATGTGGGACGACTGTAGA
TCAGCTGTCAAATCATACTTCTGCTTTTGTGGCAATTACTGCCCAACAGAAT
CTTACCCTAAAGCTTGCACTAACCCTCTCAGACTTGAAAATCTGTGGAACT
GGATTGGTCACGCTTACAAACAAACACCTTGTACAACTACTACTGGTAATG
TTTACCGTAGAACGTTTGCTGGAATCCCATCTGGAGTATTTTGTACCCAATT
TTGGGGATCCTTTTACAATAGTGTAATGGTAATCTCTGTCCTTAAAGCACTC
GGACTGAATGTTGTTGAAGATCACTTTATCAAAGTGCTCGGCGACGACGTA
ATATTCGGAACGTTAAAGCTAGTACCCATTTCGCAATGGGCCGACTTCCTCG
AAGCCTTCTCTGAAGAAGCCAAACGACGCTTCAATGCTCGCTTAAACCCTC
AGAAATGTGGCTCATCATCAGGTATACACGGTGCCCAGGTTCTAAGCTACA
CTAACTGGAATGGATATCCTAAACGAGATCCTGAACAGCTACTTGCTCAAC
TCCTCCACCCGAAGTCACTCCGTGATACATACCCAAGACTCATGGCTCGTG
CCATTGGAATCTACTACGCTAGCTGCGGTAGCCCAAAGATACGCCCGATCTG
TGAACACATTTACTCGGAGCTTAAATACGCTGGTTTCAACCCCAGCCCAAC
CGGACTCCATGGTTTATTCGATCCTAATGTTAGATTAGGATTCATTGAACTAG
ATCATTTTCCTTCGAAGACCGAAGTCATCAGCCGCTTATCTGGATTCTCAAG
AAGAGACCCTCAGCTGCAGAAGATTTACTGGAATCTCGAACACTTCTCTGA
AGAAGCTGGTTGTTGTGGAAACTGCCCCAACCAACCAATCTGAGACGATT
GTAACCCTTACTAATTTATGTACTAAGCACCATTAGAGTGCAATTTTGTACTA
TTACTAATTCTCTA

>contig-100_5990

TTTTTCCCATCTTTTAAATTCTTTTCTTTATGAAAACCAAGAAATGTCAAAT
TTAGAGTTCCTCGGACACGACCATCACTGGCCGTCGATTCCAAGATTCGC
AAAACTACCTAACGACTGGTTCTACGAATGTCAGAATCTTGTTCGAAAAA
CGATAACAGACCATGCATATACCATTGGCTTAGCCACGGCATATGATGTC
CTTAACGGATACTACAGATCCCACGCTGATGAACAGACTGCAGAACAGTT
TTTCCTCCGCTATGATGTCGAGCCTTTCGATATTATCAAAGACGAGATCTA
TTACTGGGCTATTGATAGAGTCACATTCTGGTTCAAACCAAGTCACCCTAT
CCACCCCGTCCATTTTACTGACCTAAGATGGTACCCATGGAAACTATCAAC
CAGTGCTGAACGCCCGTTCACGCACGACAATGAACTCAAATTACAGGTGC
AGCGCGCTAAACGCGAAGGCGTTATTGACAACGCTCGGATGTCATTCCAC
AATTGCTATAATCCAATATTTTGGTATTCTAGACAATATATTCACAATGTG
AAAAACGGAATTGATGTTAAGCTACACCATATTGACTTACATGTAAAACC

TGCTTTGGTTCGCACTGGCGAACCTCCTAAAGTCAGAACTGTTTTGGTGT
CCCTAAACCGCTAATATTTGCTGAAGCTATGTTCTTCTGGCCTTTATTTAG
CAATTACTTCACCGACGCTAAAACACCTTTGCTTTGGAATTATGAAACATT
GAACGGTGGATGGTATCGCTTAAACGATGAATTCTACTCTCAGTGGCACA
GCTTTACGACTATATTCAACCTTGATTGGAGTGAATTCGATATGCGAGTTT
ACTATTCAATGTGGGACGACTGTAGATCAGCTGTCAAATCATACTTCTGCT
TTTGTGGCAATTACTGCCCAACAGAATCTTACCCTAAAGCTTGCACTAACC
CTCTCAGACTTGAAAATCTGTGGAACTGGATTGGTCATGCTTACAAACAA
ACACCTTGTACAACTACTACTGGTAATGTTTACCGTAGAACGTTTGCTGGA
ATCCCATCTGGAGTATTTTGTACCCAATTTTGGGGATCCTTTTACAATAGT
GTAATGGTAATCTCTGTCCTTAAAGCACTCGGACTGAATGTTGTTGAAGAT
CACTTTATCAAAGTGCTCGGCGACGACGTAATATTCGGAACGTTAAAGCT
AGTACCCATTTCGCAATGGGCCGACTTCCTCGAAGCCTTCTCTGAAGAAG
CCAAACGACGCTTCAATGCTCGCTTAAACCCTCAGAAATGTGGCTCATCA
TCAGGTATACACGGTGCCCAGGTTCTAAGCTACACTAACTGGAATGGATA
TCCTAAACGAGATCCTGAACAGCTACTTGCTCAACTCCTCCACCCGAAGTC
ACTCCGTGATACATACCCAAGACTCATGGCTCGTGCCATTGGAATCTACTA
CGCTAGCTGCGGTAGCCCAAAGATACGCCCGATCTGTGAACACATTTACT
CGGAGCTTAAATACGCTGGTTTCAACCCCAGCCCAACCGGACTCCATGGT
TTATTCGA

>contig-100_5676

TTAGAGAAAAACTTACTTTTGGTCTTATATATTTAATGAATTTTAGTCCCTGT
AGGTAATTTAACTACATTAACACTTATTATGAAGAATATCGAGATTATTGGCT
GCAAGCCTTCGCTTGCTAACCCTATCCGTGGTAATATCGACCCCGAGTCGA
ACGTACACTACGGTAACATCGTCGATTATGCTTTAAGAAGATATTTGAACGC
TGACGAGTTCGACAAAGTTGTCAACGGCTACCGCCGATCCCAATGGGATCC
TAATGGTTTAGAAGCTGACCTGATCAAACTTGATTCAGATTATTTTGATGTT
GTCAAAGATGCTCATTATTATCGAGCAATTGAACACACCAGAAAATTGTTC
AAACCTGATACTCCCCTGAAGCCAATTCATTTCTCTGATTTACGTCACTATC
CATGGCAGTTGTCAACTAGCATTGGTGCTCCCTTTGCTACTAGTGATGACTG
GAAAGATTATGTCAATGAGAAGTATGAAAGTAATTTTACTAAATCATACTAT
CGCGATTTATTTCGTGAAGCTCACGGCATTAGTTTACTCCCCACTATGGTTG
ATCGTCGCATGACGAAACGTAACCTTTACAACGAAATGTTCTTCATTAATCG
AAAGAATATACATCTAATAAAAGATGGTCACAAATCAAATGAAAATGGTCAT
GATCTTAAATATTGGAATACTGCTTTTGCTAGACAGCATTTGGTAGAATCCCA
TGAAGAAGATAAAATCCGACTAGTTTTCGGTGCTCCATCTACATTTTTGATG
GCTGAACTTACTTTTATCTGGCCTTTACAGATTAGCCTATTAGCTAGAGGAG
AAAACTCTCCAATGCTATGGGGTTATGAAACCACTACTGGTGGTTGGTCCA
GACTGTACAAATGGGCGAACAAAACTGTTCCCCAATGTAATTTTGTAGCTA
CTTTAGACTGGAAACGGTTTGATAGAGAAGCTAGACATACTGTAATTTCTG
ACATTCATCAGTTGGTCATGAGAACTTATTTTAACTTTTCTGATGGCTATCAT
CCCACGGTCCATTACCCCGATTCAACTGGAGCTAATCCAAGAAGAATTGAA
AATTTATGGAATTGGATGACTGATGCAACACTAACTACACCACTAATGTTAC
CTAACGGTGATATATTAAGATTTAGACATTCTGGTATTTATTCTGGATATTTCC

AGACACAGATATTAGATTCAATGTACAATTGTGTCATGATCTTTACCATCTTA
TCCAAGATGGGCTTCGATCTTGAGAGAAT

>contig-100_4525

GGGACGCCGATAAAGTGTGTTTGTGGCCAGTGCTTCTCAGTTACTAAGTCG
CGGACTGGTTCTTGCAAGAGACGAACAGTTTCAAAATAGGTTGGAAAATG
AGATATATCTATCTCAGCATAACCTGGAACATAGTTCTGTCTATAGCGTAATC
CACCTGGTAAACCATGGGGATCAGGGCTATATCCTCCTGCTTTCAGGAAATT
ATAGATGTCCTCGCAAATTTGGTATACACGAGTGTGGTTTCCACAGTTAGCA
TAAGCAATACCAATGGCGCGAGCCATTAAGGCCGGTAGTGAGACCGTCCTT
TCGGGATGCCTTAACATAGCTAAAAGTTGAAGTTCGTCACGGTACGGCATA
GTAGCGTGATTCCGATACCTCAGCACTTCAGCATTTTCGAGACTTGGTAAGA
TTTCACTCTTATCAATGCTCAAAGTTGAGTTAAAATAAGCTTTCGCATAATG
AGCGAAAAACCGTAGAAATGAGGGCTTGAGAGTTTCATATAAATAAGCCAT
TAAAAGAATGGAGTCATCTCCTTGTACTTTTATAGCTATTCTCTCAAGATCG
AAGCCCATCTTGGATAAGATGGTAAAGATCATGACACAATTGTACATTGAAT
CTAATATCTGTGTCTGGAAATATCCAGAATAAATACCAGAATGTCTAAATCTT
AATATATCACCGTTAGGTAACATTAGTGGTGTAGTTAGTGTTGCATCAGTCAT
CCAATTCCATAAATTTTCAATTCTTCTTGGATTAGCTCCAGTTGAATCGGGGT
AATGGACCGTGGGATGATAGCCATCAGAAAAGTTAAAATAAGTTCTCATGA
CCAACTGATGAATGTCAGAAATTACAGTATGTCTAGCTTCTCTATCAAACCG
TTTCCAGTCTAAAGTAGCTACAAAATTACATTGGGGAACAGTTTTGTTCGCC
CATTTGTACAGTCTGGACCAACCACCAGTAGTGGTTTCATAACCCCATAGCA
TTGGAGAGTTTTCTCCTCTAGCTAATAGGCTAATCTGTAAAGGCCAGATAAA
AGTAAGTTCAGCCATCAAAAATGTAGATGGAGCACCGAAAACTAGTCGGAT
TTTATCTTCTTCATGGGATTCTACCAAATGCTGTCTAGCAAAAGCAGTATTC
CAATATTTAAGATCATGACCATTTTCATTTGATTTGTGACCATCTTTTATTAGA
TGTATATTCTTTCGATTAATGAAGAACATTTCGTTGTAAAGGTTACGTTTCGT
CATGCGACGATCAACCATAGTGGGGAGTAAACTAATGCCGTGAGCTTCACG
AAATAAATCGCGATAGTATGATTTAGTAAAATTACTTTCATACTTCTCATTGA
CATAATCTTTCCAGTCATCACTAGTAGCAAAGGGAGCACCAATGCTAGTTG
ACAACTGCCATGGATAGTGACGTAAATCAGAGAAATGAATTGGCTTCAGGG
GAGTATCAGGTTTGAACAATTTTCTGGTGTGTTCAATTGCTCGATAATAATG
AGCATCTTTGACAACATCAAAATAATCTGAATCAAGTTTGATCAGGTCAGCT
TCTAAACCATTAGGATCCCATTGGGATCGGCGGTAGCCGTTGACAACTTTGT
CGAACTCGTCAGCGTTCAAATATCTTCTTAAAGCATAATCGACGATGTTACC
GTAGTGTACGTTCGACTCGGGGTCGATATTACCACGGATAGGGTTAGCAAG
CGAAGGCTTGCAGCCAATAATCTCGATATTCTTCATAATAAGTGTTAATGTA
GTTAAATTACCTACAGGGACTAAAATTCATTAAATATATAAGACCAAAAGTA
AGTTTTTC

>contig-100_19074

GTATTTACCGGGTTGGGGCTTGGGAACTGAGATGCCGGAGGAGAACGCCG
ATGCCTTGCTGGCACTAGCTGCCTGACCGGGTTGAGAACCAACCGCTGATC
GACGAGCTTGGCGTTCAGCCTTACCAGGCTTAGACTTACGTCCAGCCGTGG
AGGCGGAGTCGCTGGGGGCGACGGTAGAGGGGAGTTGCGACTGGGTGTC

AGCCATGGCCGCAAAGTGAAGGAGGCAGGGGGGGGTGTTATATTCTTGGA
GGCTTGAAAGGCTTAAATATAACGAGAAACACCAGATAAACTGGTGAAAA
GACAAG

>contig-100_19083

GCTGGGCCAAACGCAGGAGAGCAGCGACTACGAGATCCTTGGTGAACTCA
TGGTCGTCTATCTCAGCATGAGACTTAAACTCTGCATACTTGGCGTTGACCG
TAAACCGATCCGGAAAGGTCAGGAGCGAACGTTCGAGAACACGCTCGTCG
ATTGCAAAGGTCTGGTCCCGAGCCGGTTCACCCGCACCCGTCTGAAAAAC
GACAGAGTATTTACCGGGTTGGGGCTTGGGAACTGAGATGCCGGAGGAGA
ACGCCGATGCCTTGCTGGCACTAGCTGCCTGACCGGGTTGAGAACCAACC
GCTGA

>contig-100_5397

GTCTAGCGCTTTTACCGTGTCAACGGGTTTATGCCTCCTTCATTCCTTGAGC
GAAATGGAAGATTTTACTCAAGATCCAACAACACACAACATCGTTGCTGAG
GGGTCTCATTTAATTGACGCACTCCACCTCCGGCCCCCAAAGCAGAGGTCA
GCCACCAGCGAAGATGTTGTTTCTTCCAACTTCAGGTCTCCCAACCTGGCG
GAAATCGCAAAATACGGGGGGTACTCAACGTACTCGTCTAACTCTAACACC
GATCCTTATGTGAGAGAGACACTAAAACTATTTTCTCGAGACACCTACGAG
GATATCCGTGGTTTCACTCGTCGTCCGCAAGGGACTCCAGGCATGTACACT
GCCTTGAAGAAGTTCAGCGGAGAGCGAAACACATTCGGTGATCTCTCCCCT
AGTCAGCAGTCTTCGATGCGCCGTGCCATCGGCAAAGCGAAGAAAGCTTT
CAAACTCCCCTACAAGCGCGAGCCGCTTGATTGGCATGAAGTGGGTCAGTT
TCTAAGGCGTGATACCGCCGCAGGAGCAACCTTCATGGGCCAAAAGAAGG
GTGATGTGATGGAAGACATTTACCACGAAGCTAGGTGGTTAGGACACCGGA
TGAAACAGGATGGTAGGGCCGGTTTCGATCCAACCCGAATGAGGTTCCCTC
CTTGTTTGGCAGGCCAGCGTGGGGGTATGTCGGAGATTGACGACCCCAAA
ACGCGCCTGGTTTGGATTTACCCTGCTGAGATGTTGGTAGTTGAAGGGTTCT
ACGCCCCTTTAATGTATCGTGACTTTATGATCGATCCAAATTCACCAATGTTG
AACGGGAAGAGCGCGCAGCGTTTGTACACCGAATGGTGCTGCAAGCTAAG
GGATGGGGAAACACTATATGGCATTGACTTTAGTGCTTTTGACACCAAAGT
CCCAGCGTGGCTAATTCGCGTGGCATTCGATATTCTGCGTCAGAATATTAAC
TTTGAGACGTTCGGTGGTAAACCAGTCAAGAAGCAGGATGCTCAGAAGTG
GCGAAACGTTTGGGATGCCATGGTGTGGTATTTCATAAATACACCCATACTT
ATGCCGGACGGCCGGATGTTCCGGAAGTTTCGGGGTGTACCCTCCGGATCG
TGGTGGACCCAGATGATCGATTCAGTCGTAAATCATATCCTGATTGATTATCT
TGCGGACTGCCAGCGTGTAGGGATCCGAAACCTGAGGGTGTTGGGAGATG
ATAGCGCTTTTTGCTCTGGTGACCAGTTTGATCTGGAATTAGCAAAAGGTG
ACTGCGAGAACACCGGTATGGTGATAAAGCCCGAGAAGTGTGAACGAACC
AAAGACCCAGGTGAGTTCAAGCTATTAGGCACGACGTACCGGGGGGGCCA
CGTTTTCCGTGATACGGAGGAGTGGTTCAAACTCGCTCTCTATCCCGAGTC
CAGCGTGCTGACGTTGGATATTTCTTTCACCAGGTTGATTGGCCTGTGGCTC
GGTGGAGCAATGTGGGATAAGGAGTTCTGTGAATACATGGACTTCTTCCAA
TCAAGCTATCCTTGTCCTGAGGAGGGGTGGTTCTCCAAAGATCAGAAAAG
GTGGCTTGAGATAATCTACTCAGGCAAAGCGCCAAGAGGTTGGACTTCCA

AGAAGAGTCTCTTTTGGCGATCAATCTTCTATGCCTACGGCTAGGAGTGAC
AAATCTTCTGCAGATAGCAGAGTCTTGCGTGTATACGCGGATGTGACATTGA
CACGCCGGGGCGACGGATGGTGATCCCCCTGGCCAGTGCACGTCTGCTGT
CAGATAAAGTCTCCCGTGAACTTTACCCGGTGGTGCATATCGG
>contig-100_7674
CCCAAGGCGATTTCGCCCCCGTTGCTTCAAGCGACGTCCGTGTTCCCGCTT
CGCTAGCTGCGTTTATTTCGCAGTTTGGTGAGTTCTCCGTTCCCACTTTAGG
GACTCGGTTTTTATTCTCGGATTACGAGAATACCGTACGTTCTATCGTATGGA
TGGCCGAAGGAGTAAAGCGCAACGGAATTGGAGGCGCTCCTTTGAAGAGA
TCGTGGTTGCCCGTCCGTCCATCTGACGGGCACACAAAGACCGTGATAGCT
TCACGCCTGTGCGAGCTTCTGGCGCAAGCAGAGCTCGAAGTCAATCCTGA
CGTCCTTGAGGGGGGCGTACTTTCAGGAGAGATTCCCGATGCTTGGGAGA
GCATCAAACCCGTCCTTGGAGACGGTGACGAACGTCGAGACAGATTCGAC
TTTTTATTTAAGTCGTATCGTGATGCTCCTGCGTTCGTCACTGCCTTCACTAC
CACCAGTGCATCTGGTGTGTTGGCTGAGTTAGATCTTAGCTGGGACCGTCC
GTCCGCGGGTCACGTAGACTGGACTTTTAACCCGAAAGAAGTGTTTACTCG
CCTTTCGGATGCCTGGGCACGTAGGTCCACAACCTACGCCCTATTCTTCGA
GTTGTCGTCGAGTCAGATGACGCGTAACGTGTCAGCTGGGAGCCAGTCCC
AGATGGCGCGTGTGAACACCCGTGATGGTGTGACCGTCGTCAAGACTCATC
TTGGTTTGTCGGCCCCCGAGTTCTCACTCGTGGCATGCTTCCCCGCATCAGT
TATCTTTACTGGTGGGCTGACCCGTCGCGTGGTGGTGACCACGCCACTCTC
TGTTGAACAGAGAGCCACTGAATTCGTTCAGATGGATTGGCGCTAGTCACT
GCGGTAACGCAAAAAGACAACCTACCCAACTCAGTAGGAAAGGCAGTCGT
GTAAACGACACCGTGACTCCGTGTATCGGAGAAGTCCTGTGTATCAGGCAC
CCAACTTTTTAACAAGAAGTGGGAAACGTGACAGTTTATCTGTCGTGTGTC
CGTAAAACTCCCCCGAGTTCTCACTCGTGGCATGCTTC
>contig-100_15097
TCTATCTTTCGCTTTCAAAAGAAGAACCCCTCCCCTCACTTCCACTGGAATA
TGCGCATACCCCCTTCCCTCAGGGGTTTGCGTCTTATACTCGGGTGGCAGCG
GGTCTTTCGAAGGGGGCCTGGCGTCGGGCCCGGGTCGTTGAGGGTTCCTT
CCACCTTGCGTGCAGTTTGTATGCCCAGATGCCCGTCCGCCCCCCTTCTTGC
GGGGACTGGCGGGCGGTCCTCCAGCGCGGCACGCCTCGCTTCTGGGAGCC
CAGATCTTGTATCCGCAAGTTGTTCGAACAAATTTGGCGGAGACTCTGTGG
CCCTTTTGCGCAGGTGGTGGGGGCAGGAAGGACGGAGGAGAGGCTGATGT
TGCCGGTCGGGGAGGAAGAAGAGGTACAAAG
>contig-100_15987
GAGTCTTGCCCTTGGACAAGACTAGGCCGGAACCCACAACCCCATCCGCC
CACTTCTCTGCCTCCTCAGGACGACAACGGAACACAATGTCGTCCCCGTGC
ACGCGGACTGGAACTCCCCTACGGGGAACCAGCCAGCGGAAGACGAGGT
AATTAACGAGACAGAGAAGAGGGAAGGATATGAAGGTCCCCATCAGCTGA
CCGCGTCGCTGCGGTGCGCACAGTTTACTTGTGTACAAGAAGGCTTGCGAC
CTTGCCAAGGCAAGGGCCCAAACCGACTTAGGTACACAGGGAGAGAGGCT
CGAGAGGACTCGCAAGACGCACTGACTAACTTCCATCGATAAGTTATCAGT
AGCCGCCTCGTAGT

>contig-100_17699
GGCCGGAACCCACAACCCCATCCGCCCACTTCTCTGCCTCCTCAGGACGAC
AACGGAACACAATGTCGTCCCCGTTCACGCGGACTGGAACTCCCCTACTG
GGAACCAGCCAGCGGAAGACGAGGTAATTAACGAGACAGAGAAGAGGGA
AGGATAGGAAGGTCCCCATCAGCGGACCGCGTCGCTGCGGTGCGCACAGT
TTACTTGTGTACAAGAAGGCTTGCGACCTGGCCAAGGCAAGGTCCCAAAC
CGACTTAGGTACACAGGGAGAGAGGCTCGAGAGGACTCGCAAGACGCAC
TGACTAACTTCCATCGATAAGTTATCAGTA
>contig-100_19940
GAAAAAGGAAATACCGCGCTTGTTCCCCCGCGGTTGGGATCGCCACTATCG
CTCCAGAGCGGGTGGTCTTCTTCTTCCTGTGAAGTCCTGCCTTGAGGCAGG
TCGTAGGAGAGGTGGAGGTCGTTACCGGAATTCGCAGCTCCGGTACCTCCG
TCGTGTTGCGCTAGGCTGGGAGACTGTGGAGTCTCCCGACGCCCGGGCGC
GGGTGACATGTGTTCCGGACGGGTGTAAACTCCGTACGGTGACTGTGTCAT
CCGTGGACCAGGCGGCTCTTAAGCCCTTGCACGAGACCCT
>contig-100_20798
ATCAGCCTCTCCTCCGTCCTTCCTGCCCCCACCACCTGCGCAAAAGGGCCA
CAGAGTCTCCGCCAAGTTTGTTCGAACAACTTGCGGATACAAGATCTGTGC
TCCCAGAAGCGAGGCGTGCCGCGCCGGAGAACCGCCCGCCAGTCCCCGCA
AGAAGGGGGGCGGACGGGCATCTGGGCATGCAAACGGCACGCAAGGTGG
AAGGAACCCTCAACGACCCGGGCCCTACGCCAGGCCCCCTTCGAAAGACC
CGCTTCCACCCGAGTATAAGACGCAAACCC
>contig-100_20854
AAGGCTTGCGACCTTGCCAAGGCAAGGTCCCAAACCGACTTAGGTACACA
GGGAGAGAGGCTCGAGAGGACTCGCAAGACGCACTGACTAACTTCCATCA
ATAAGTTATCAGTAGCCGCCTCGTAGTCCCCGGAGACATAAACCTCCCCTTT
TCGCAGGCCGAAAGAAGTAAACTGCCCGTGCACAGCGTCACCGCGGAGTA
GCCAGTCCTGAGTTGAGATGTGGTCATAGAGGGTCTCGTGCAAGGGCTTAA
GAGCCGCCTGGTCCACGGATGACACAG
>contig-100_29403
GGCGGCTACTGATAACTTATCGATGGAAGTTAGTCAGTGCGTCTTGCGAGT
CCTCTCGAGCCTCTCTCCCTGTGTACCCAAGTCGGTTTGGGACCTTGCCTT
GGCAAGGTCGCAAGCCTTCTTGTACACAAGTAAACTGTGCGCACCGCAGC
GACGCGGTCAGCTGATGGGGACCTTACTATCCTTCCCTCTTCTCTGTCT
>contig-100_11639
GGGTGCCAACCTGCAGATTCCTCTTATGTGGCAGCCCGTTTATGATCGTTTG
GTTGGTCTGGCGCCGAACGTCGCAGCCATGTCTTCTGGTGTCACTGGTGCT
CTTCGCGCTCTGCCCCCCTTCGTGCAGTTTCCGAACATCCAGGTCCGCCCT
GATGTTTTGCAGTACCAGTTCACGGCTGCTGATCGCGTTCTTGCCCGCCAC
CTGGCTTCCGACCTTAAGCAGTATCCCCAGGTTCTCAACTACCGCGAGGTC
GGTGGTCCCTCTGTCGACAACCTCACTCACTCGTTGGCTCAGGAGGTGAA
GTACTCCCCTCTCCCCTCCGTTGAGGTCGTCCTGCTCAACGGTGCCCCGGG
TTGCGGTAAGAGTTTTTGGCTCCGTAACGCGGTCCGTGCGTTTGAGGCTGA
TGGCCCGGTGCGCTTTCACACCTGGAACAATCGCCTTCGTGTCGACATTGA

GCGTACCTTTGAGCCTATTCTGTCGTATTGGGACGATCGCTCCGCTTGTTCT
GGCATGACCCCTCTTTTCCAGAAG

>contig-100_9446

CCGGGTTGCGCCGGGGGAAGGGGGCTTGTTCCCATCACAGTGTGTTTTTC
TCACTGCTTCACGGAGCGCGGTACCCCCACCTTCACCGCCGTTGAAGGATC
GGGACACTAGCACGATGGAGTGCACCAGCCCACTTTAACTGGCCACCGTA
CCTAATAACAGGGTGTCACCCTGGACCTTCGTGGGTTTTAGGAGAACAAGA
GAGCGGAGGTTACAACCAGCAGCCAGGCTGGAACCGGGTACGACACCGGT
AGCGGTCTTAAGGGGAACCGAACCCACGGACGCCACGCAAGGTGGCAAA
AGAGGGAGAGGAAGGGACGGGAGAACAAAGACAGAGAGGGCAACGAAA
CGGAAGAAGAAGGAGGGGAGCTAAACAACACGGGGGGGATAACGGGAGG
GAGGAAGGTGGAAAAGCTCACGGGCTGACAAAGAGACGGCGACGGCAGA
CGAGAGGACGAGGGAAGGAGTGAAAACATCGTCTGAGGCAAAACGCAAT
GACAAATCGAAAGAATCCCAGAAGTCGGCGTCGGAGCGACCATCTTCAAG
CGCGAGGGTACCCCGGTGCAGCAAAACGTCGGGAGAGATGTGAAGAGAG
TGAAACCCGTAAACAAAGCCACAAAACTCACCCACATCACCCACGCCCAG
CTTCGGAGAGAAAGGGTACGAGTCGGAGAGGACATGATCAAAGCGGCCGC
AGAGGATCATGTCGTCACCAGAAAAGCAGGCGGGGACGTCGG

>contig-100_10957

CTAACCGTGAGAAGCCCGCTTTCCAGGGTCGCCTTCTCAATGTCCAGCACG
CCTTGCGCCACCAGCTGCCTGTCCCCGCTGATACTGTCCTTGTTCGCCCTCG
TCTTCCCGCGGTAGTCCCGGATGCTGTCCCTGTTGGTCTCCCCGACCCTGTT
GTCGGCGCGCACCCTGACCTCCTCCCTGAGTCTCGTGAGTGGTTCGTGTCC
TCGGTCGGCTACACGCAGCAGGTGTCGCTCAATGGCTCCCCTCTCGGCCTG
CATCATACCAGGCGCGATGCTGCTACTGAGCAACTCTCTTTGGCGGAGCGG
ATTCGGCCTCAGGTCCCGCTGGTTCGTGCGCGTCATCGTCGTCAGGCTGCT
GACTTGCGTCGTGGACTCTCCTCCTTTTTGTCGCTTGGTGGCTACGCCTTCA
AGTTCGGTCTTTACCAGGATTGCCTGGTTGAGCAGCTTGAGTCCTGGGTTT
CCGGTCGTACCCGTGCTGACATTGAACGGGCAGTGGCCAACTCCCCCCCTG
ACTGGGATCGTCTGTTCGTCCGCCTGTTCCTGAAGTCCCAGCGCGTTAAGA
AGATTGCCAAGCGTCATGCGCCTGCTGGTAAGGGCCAGATCGTCACCGATG
TCTCCCATGTTAAGCTGTTCGGTGATGCTTTCTGGGCCCTGTACCTCGAGCG
GTCTGTGCTGCGTTCCCTTCGCCCCGGTTTCTGTCTGCACACTCGCTCCAAT
GTCACCGCTATGCGCTCCTG

>contig-100_6649

CGAGCGGTCTGTGCTGCGTTCCCTTCGCCCCGGTGTCTATCTGCACACTCG
CTCCAGTGTCACCGCTATGCGCTCCTGGTACTCCCGTTGGTGGCGTCCTGG
TTCTGGCGTCACCTGGTGCGACTACACCGGGTGGGATTCCGGGGTGAACG
AGTCTTTCACCTTGCTCTACACTGATGTCCTCCGCTCGTACGGTGTCCCCTC
CGCCGTGGCTGCGCGTTTCGCCTCTGACCGCCATTCGCTCCGTTCCTTTCTG
GGCCCGATGCCCGCCATGCAGGCGTCTGGTGATCGTTACACTTGGCTTGCG
AACACCCTCGGTAATATGGCGCTCACTGGGATCTCGTTTGACTTGCCCCCC
GACGTCCCCGCCTGCTTTTCTGGTGACGACATGATCCTCTGCGGCCGCTTT
GATCATGTCCTCTCCGACTCGTACCCTTTCTCTCCGAAGCTGGGCGTGGGT

GATGTGGGTGAGTTTTGTGGCTTTGTTTACGGGTCCCACTCTCTTCATATCT
CTCCCGACGTTTTGCTGCACCGGGGTACCCTCGCGCTTGAAGATGGTCGCT
CCGACGCCGACTTCTGGGATTCTTTCGATTTGTCATTGCGTTTTGCCTCAGA
CGATGTTTTCACTCCTTCCCTCGTCCTCTCGTCTGCCGTCGCCGTCTCTTTG
TCAGCCCGTGAGCTTTTCCACCTTCCTCCCTCCCGTTATCCCCCCCGTGTTG
TTTAGCTCCCCTCCTTCTTCTTCCGTTTCGTTGCCCTCTCTGTCTTTGTTCTC
CCGTCCCTTCCTCTCCCTCTTTTGCCACCTTGCGTGGCGTCCGTGGGTTCGG
TTCCCCTTAAGACCGCTACCGGTGTCGTACCCGGTTCCAGCCTGGCTGCTG
GTTGTAACCTCCGCTCTCTTGTTCTCCTAAAACCCACGAAGGTCCAGGGTG
ACACCCTGTTATTAGGTACGGTGGCCAGTTAAAGTGGGCTGGTGCACTCCA
TCGTGCTAGTGTCCCGATCCTTCAACGGCGGTGAAGGTGGGGGTACCGCGC
TCCGTGAAGCAGTGAGAAAAACACACTGTGATGGTAACAATCCCCCTTCCC
CCCGGCGCAACCTGGGCCGCATTAGAAGGAGCCGAAATCCGTTAATTCCGT
CCCATTCTCGATCATGGGATGTCACGCTGCGGAGCGTGGCTGTGCAGAGGC
CGTACGGACACCGGCACTTCCTCGCGCTGTAAGACACGGTCCACAACACC
TCATTCAACTGAGGCTTCAAGATCGCTTCCCTTTCTCACACTTTTCCTTGTT
TGCGTTTGTTTGTTGCGTTTCCTCATCCTCTCTGTTCCCTTTCTATTGTTATGT
TGTCTCCCACCGCCTCCCTTGGGATCGCTCTCCTTGATGTTGATTTGGTTGA
TCTTGATCGGGCTCGCCTCCTCTGTGAGCTTGATCTTCGCGTGGCTCAGGC
CGCGTTGTCCGAGGTTCGTGCTCGTTTCCCCCCCCCCCCCTCCCCCCCCCC
CCCCCCCCCCCCCCCCCCCCCCCCCCCCCCCCCCCCCCCCCCCCCC

>contig-100_6965

CATCAATCGAAAGACCCTGAGCGTCGACAAGGGGGTACGTGGGGGTGCCA
CCCTTGGACTTAGACTCGGCGAACCGGGGAGAGGTGACGAGCAGCGGGA
CGTCCCGAGGCGGATTGGAAACCACGACAACAGCACCATGGGACACCTGA
GCACCCACGTCGAGGGCGCGGGGCAAACCGAAGAGCGCAGAATTCTCAAT
CGACAACCGGCGATTCACACACGAGTAAGAGGTGGAAAGGGAATTCAACC
AATCACAAGTGGGGGGGTCGGAACGGGAGAGAGAGTTGGCCTCAGGAAA
GGGAGGGTGAGCCTGCGAAGAATCGAACGTGAAGACCATACGCCGAAGA
CCGGGGCTGGTGAGCGCGATCATCTGGATCATACCGGGGTAAAGAAGGGC
GGCATCATCGAAGACCAAGGTACCGGAGCTCTTCTGGAAAAGAGGGGTCA
TGCCAGAACAAGCGGAGCGATCGTCCCAATACGACAGAATAGGCTCAAAG
GTACGCTCAATGTCGACACGAAGGCGATTGTTCCAGGTGTGAAAGCGCAC
CGGGCCATCAGCCTCAAACGCACGGACCGCGTTACGGAGCCAAAAACTCT
TACCGCAACCCGGGGCACCGTTGAGCAGGACGACCTCAACGGAGGGGAG
AGGGGAGTACTTCACCTCCTGAGCCAACGAGTGAGTGAGGTTGTCGACAG
AGGGACCACCGACCTCGCGGTAGTTGAGAACCTGGGGATACTGCTTAAGG
TCGGAAGCCAGGTGGCGGGCAAGAACGCGATCAGCAGCCGTGAACTGGTA
CTGCAAAACATCAGGGCGGACCTGGATGTTCGGAAACTGCACGAAGGGGG
GCAGAGCGCGAAGAGCACCAGTGACACCAGAAGACATGGCTGCGACGTT
CGGCGCCAGACCAACCAAACGATCATAAACGGGCTGCCACATAAGAGGAA
TCTGCAGGTCGGCACCCATATCAAACCAGGAGACACGGACGGAAGAAAGG
CGCACTAGGAGGTTGGGGAGCGGAGGCACCACAGCAGCGCCAAACTGGG
GGAGGCCAAAACGAAGGTGAAGGTCGGCGCCAGCGCCGTAAAGGGAGGA

GGAGCCAGAAGGATACGTGGCCCGAGCCGGCTGCACGAAAAGAGGGGGA
GCGCCGAGCTGCGCCTGAGGCAAGCCGTTACCAAGAAGCGCGGCTGAGTA
AATCGTAAGCCCGACCTGGAAATAGGCGAGGACCCGGGGCAAATCCTGCA
AAGCCACAGTGCCATCATCAAACGCCGCGAAGGCAGCGGGGGGAACGGA
CGAAAGGAAAGCGGCCAAGCAAGTGAGGGGCGGCAAGCCAAGGAGCGC
ACCAAGGCAATCCCAAACACGGATCCGAGCAGGGTCAGCCCTGGGAATGA
AAGGCACCGGGAGACGGAGAAGAGCGCCGCGCCAGAGGGGGAAATCAAG
AGTGGCCGGG
